# Diversity and traditional knowledge of medicinal plants used by Shui people in Southwest China

**DOI:** 10.1186/s13002-023-00594-4

**Published:** 2023-05-30

**Authors:** Sizhao Liu, Beixi Zhang, Qiyi Lei, Jiangju Zhou, Maroof Ali, Chunlin Long

**Affiliations:** 1 Key Laboratory of Ecology and Environment in Minority Areas (Minzu University of China), National Ethnic Affairs Commission, Beijing, 100081 China; 2 Key Laboratory of Ethnomedicine (Minzu University of China), Ministry of Education, Beijing, 100081 China; 3 School of Ethnology and Sociology, Minzu University of China, Beijing, 100081 China; 4 College of Life and Environmental Sciences, Minzu University of China, Beijing, 100081 China; 5 School of Health Science, Kaili University, Kaili, 556000 China; 6 Guangxi Subtropical Crops Research Institute, Nanning, 530000 China; 7 Center for Integrative Conservation & Yunnan Key Laboratory for Conservation of Tropical Rainforests and Asian Elephants, Xishuangbanna Tropical Botanical Garden, Chinese Academy of Sciences, Mengla, 666303 China; 8 Yunnan International Joint Laboratory of Southeast Asia Biodiversity Conservation, Xishuangbanna Tropical Botanical Garden, Chinese Academy of Sciences, Menglun, 666303 China; 9 Institute of National Security Studies, Minzu University of China, Beijing, 100081 China

**Keywords:** Medicinal plants, Traditional knowledge, Ethnomedicine, Shui ethnic group

## Abstract

**Background:**

The Shui are a small Chinese sociolinguistic group living in Sandu Shui Autonomous County, south of Guizhou Province. The Shui people have accumulated and developed rich traditional medicinal knowledge, which has played a significant role in their healthcare. Traditional ethnic herbal medicines, like Shui ethnomedicine, have become an important resource of rural development in Guizhou Province. However, not much research has been conducted to document the medicinal plants traditionally used by the Shui people. This study’s aims are (1) to record the current use of medicinal plants in Sandu County and associated traditional knowledge, including the medicinal plant species used and the types of diseases treated by local healers and any unique aspects of their preparations; (2) to analyze the most important medicinal plant species using relative frequency of citation (RFC); and (3) to provide useful information and data for possible future development and application of ethnomedicine and promote the conservation and preservation of Shui traditional medicinal knowledge.

**Methods:**

Field surveys were conducted between July 2015 and August 2022 in Sandu County. A total of 15 local healers as key informants were interviewed. An additional 132 informants from villages and local markets were also interviewed through semistructured interviews and focal group discussions. Local Shui healers were followed during their collection of medicinal plants in the fields and recorded the medicinal plants’ names, uses, and parts used. An ANOVA was used to evaluate the number of medicinal plants recognized by local healers across age-groups and townships, and relative frequencies of citation values were determined for the recorded medicinal plants.

**Results:**

In this study, data collected from 15 Shui healers and 132 other informants were analyzed. The healers used fresh or dried parts of 505 plant species as medicine to treat a wide range of conditions and diseases. These plants belong to 405 genera from 156 families, with Fabaceae being the highest represented plant family. The Jiuqian township had the highest distribution of per capita healers (pch); only one local healer was in Zhonghe. Of the 15 local healers, only two were younger than 40 years of age. There is a major concern that traditional medicinal knowledge may be lost if there are not sufficient trainees or suitable successors. Among the common medicinal plants, most are herbaceous and the Shui typically use the whole plant in their medicines. There are 85 different recorded diseases treated by Shui medicinal plants, and among them, rheumatism and bone fractures have the largest number of species used. Three medicinal plant species, *Isodon amethystoides*, *Asarum insigne*, and *Acorus tatarinowii*, are the most commonly used ethnomedicines by the Shui people.

**Conclusion:**

This study demonstrated that Shui people have extensive knowledge of a diverse range of medicinal plants, many of which had not been systematically recorded before the current study. Further research on the chemistry, pharmacology, and toxicity of Shui medicinal plants will be useful for developing functional foods or pharmaceutical products, particularly those of *Isodon amethystoides*, *Asarum insigne*, and *Acorus tatarinowii*. Additionally, as a result of rapid economic growth, fewer young people in Shui communities pursue traditional medicinal studies. Only 15 traditional Shui healers remain in the county, and only two of them are below the age of 40 years. Therefore, to conserve Shui’s traditional medicinal knowledge, initiatives and policies are required to regenerate, strengthen, and promote Shui medicinal knowledge.

## Background

Traditional medical systems worldwide have a long history of preventing and treating diseases while supporting community health [1]. As such, traditional medicinal plants have been studied both for conserving ethnomedicinal knowledge [2] and for modern drug discovery [3]. Local communities throughout China have maintained and transmitted rich traditional medicinal systems over centuries, including traditional Chinese medicine, as well as a diverse range of ethnomedicinal practices of the numerous minority sociolinguistic groups of the country [4]. Guizhou Province is one of China’s four major medicinal production areas. It is known as “Western China’s Medicine Center” due to its rich ethnomedicinal resources, including those of the Shui communities [5].

The Shui people, a sociolinguistic group residing in Sandu Shui Autonomous County, are situated in the south of China’s Guizhou Province. The Shui population is around 410,000 [6]. With a long and storied history, the Shui people have amassed a wealth of folk medicinal knowledge, detailing numerous distinctive treatments for local common ailments such as traumatic injuries, bone fractures, and snake bites [7]. This repository of Shui folk medicine has been continually enriched and refined through generations of practical applications. For example, the Shui people's practical need for utilizing herbal medicine to prevent and treat diseases has fostered the development of their distinctive medicinal plants market customs during the Dragon Boat Festival.

According to previous studies, there are more than 300 common medicinal plant species combined in numerous medicinal formulations by the Shui people [8]. Some publications related to Shui medicine include “Shui Nationality’s Medicine” [9], “The Treasury of Knowledge of Medicine of Shui in China” [10], “Summary of Ethnomedicinal Plants in China” [11], and several papers [12–14]. The local traditional Shui medicinal knowledge and managing experiences which are practiced, accumulated, and passed down from generation to generation may play a significant role in the sustainable use and development of Sandu plants resources.

In previous publications about Shui medicinal resources [15–19], traditional Shui medicinal plants and ethnomedicinal knowledge have not been systematically reported. Normally, the practice of traditional medicine is handed down through generations, and the old generation who hold the traditional medicinal knowledge must impart it to the next generation of healers before they die. However, nowadays few younger Shui people elect to learn traditional medical practices. Thus, the traditional knowledge regarding herbal medicines in Sandu should be documented immediately to avoid becoming endangered in the near future.

This research focuses on understudied Shui medicinal plants and associated traditional knowledge in Sandu County, with the following three aims: (1) to record the current use of medicinal plants in Sandu County and associated traditional knowledge, including the medicinal plant species used and the types of diseases treated by local healers and any unique aspects of their preparations; (2) to analyze the most important medicinal plant species using relative frequency of citation (RFC); and (3) to provide useful information and data for possible future development and application of ethnomedicine and promote the conservation and preservation of Shui traditional medicinal knowledge.

## Methods

### Study area

**Fig. 1:**
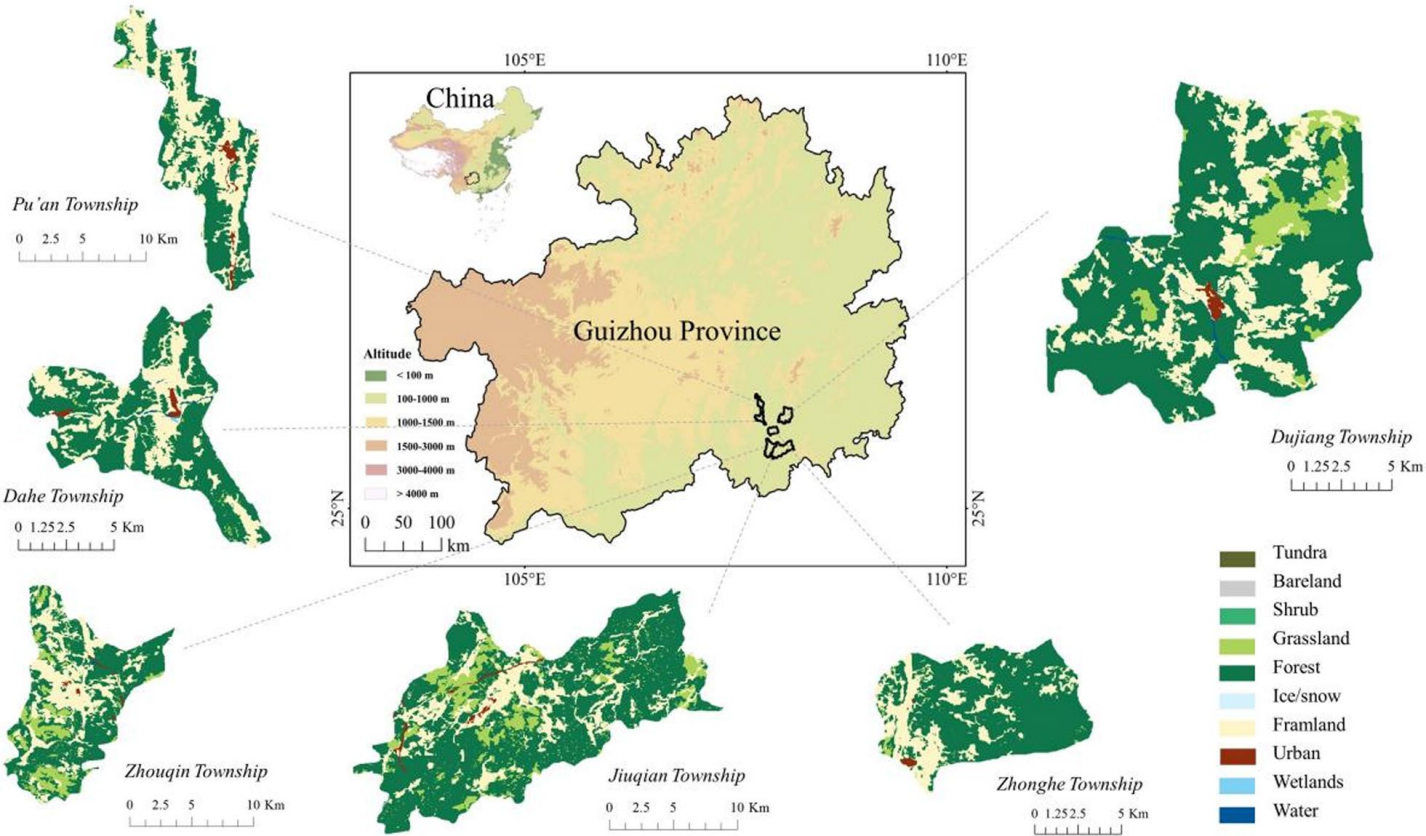
Sites for field surveys in Sandu County

The study area is situated in Sandu Shui Autonomous County, a part of the Qiannan Buyi and Miao Autonomous Prefecture in Guizhou Province, Southwest China (Fig. 1). Located between the Moon and Leigong Mountains, the study area spans 25° 30′–25° 10′ N and 107° 40′–108° 14′ E. Sandu County stretches 56 km from east to west and 78 km from north to south, encompassing a total area of 2380 square kilometers [20]. This region features a low mountainous landform type, with Gengding Mountain in the northwest as its highest point at 1665.5 m above sea level. The lowest point is positioned at the terminus of the Duliujiang River, with an altitude of 303 m, while the average altitude is 675 m. The area’s climate is classified as subtropical humid monsoon, characterized by extended summers and brief winters. The complex terrain, topography, and altitude variations have allowed the county to preserve a wealth of medicinal plant resources, which support the Shui people and their medicinal practices [21]. Preliminary botanical surveys indicate that there are 736 species of angiosperms in this area, and the main timber species include *Cunninghamia lanceolata, Pinus massoniana*, and *Phoebe zhennan* [22].

### The Shui people

In Sandu County, the Shui people account for 65.93% of the total population, and the remaining consists of Han, Buyi, Miao, and Yao sociolinguistic groups [23, 24]. The historical origins of the Shui ethnic group date back to the period before the Qin and Han dynasties. They were once part of the “Luo Yue” group, which itself belonged to the larger “Bai Yue” collective of ethnic groups inhabiting the ancient Lingnan region. Due to conflicts and wars, they migrated from the Yongjiang River watershed area, traversed Hechi and Nandan, and ultimately settled near the Guizhou and Guangxi border [25]. Over time, they gradually diverged from the “Luo Yue” group and established their own distinct ethnic identity.

The traditional culture of the Shui people still retains many elements of the ancient “Luo Yue” culture. For example, they inhabit elevated wooden structures known as Ganlan-style buildings [26]. These buildings have a dual-purpose design: the lower section serves as an area for livestock and storage of farming tools, while the upper portion is dedicated to human habitation.

The Shui language belongs to the Kam-Shui language grouping within the Sino-Tibetan language family [27, 28]. The Shui people possess their own written language, using the “Shui characters.” However, with just over 400 characters, it is a limited medium for exchanging ideas and is mainly employed for ritualistic purposes, such as worship ceremonies [29]. As a result of their long-standing interactions with the Han Chinese, nearly all Shui individuals are now proficient in both the Shui and Mandarin Chinese languages. Consequently, Mandarin has become the predominant written language in their everyday lives.

In the Sandu Shui Ethnic Autonomous County, generations of Shui people have thrived amidst the high mountains, dense forests, and warm climate. However, due to historical transportation challenges, modern medical resources are often scarce [10]. Through long-term struggles with illnesses, the Shui people have gained extensive experience in using local herbs, which they call “hama,” to prevent and treat various diseases. Within the Shui community, it is common for individuals to be familiar with several medicinal plants, leading to the accumulation of numerous medicinal prescriptions for disease prevention and treatment.

### Field surveys

**Fig. 2:**
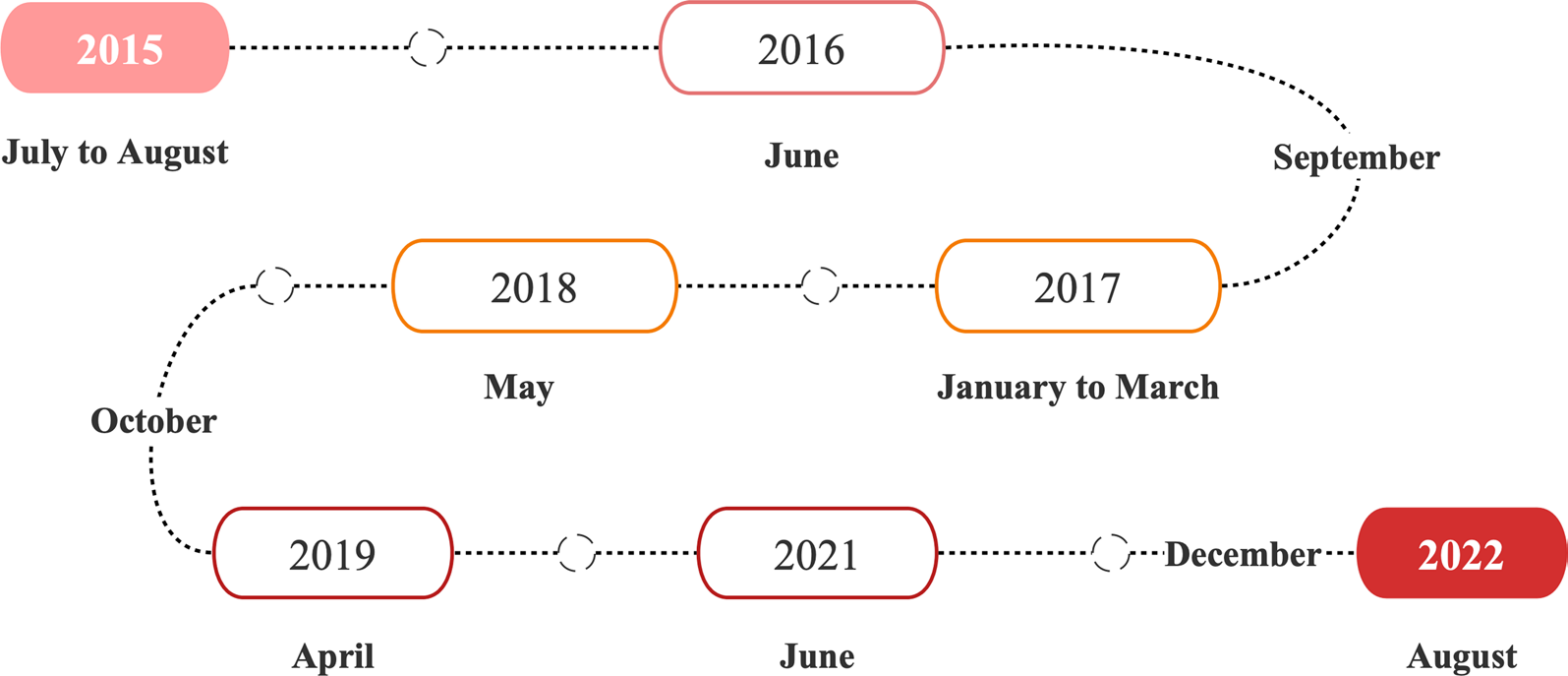
Field surveys conducted from 2015 to 2022

Ten field surveys were carried out from July 2015 to August 2022 (Fig. 2), which lasted for up to 3 months in total, using participatory rural appraisal (PRA) and semistructured interviews, participatory observations, and focal group discussions in the investigation sites [30–33]. The species, habitats, and varied uses of Shui medicinal plants were collected and documented. The field surveys were carried out in six townships, including Jiuqian, Pu’an, Zhouqin, Dujiang, Dahe, and Zhonghe. The field sites were identified after researchers observed and talked with suppliers in the open markets where medicinal plants were sold.

In the Shui region, most individuals have some familiarity with herbal medicine. However, only a select few individuals are recognized as dedicated Shui healers who exclusively practice this profession. The identified Shui healers were interviewed as key informants for this study. These key informants are renowned local healers with rich medical experience and effective treatment outcomes, serving as vital custodians and contributors to indigenous medicinal knowledge. In addition, other members of the Shui community, who possess only basic knowledge of herbal medicine and do not derive their livelihood from it, were regarded as additional informants. They contributed extensive ethnobotanical data to enrich the information gathered during this investigation.

A total of 15 Shui healers were interviewed as key informants, including 2 females and 13 males. Each of the Shui healers has practiced traditional medicine for more than 25 years. They have had rich medicinal experiences and positive clinical outcomes and are recognized in their communities as important custodians and practitioners in the knowledge of traditional medicine. Interviews with key informants included discussions regarding diseases, compatibility of medicinal materials, processing and treatment methods, taboos, and means of a succession of information. In this study, local Shui healers were followed during their collection of herbal medicines in the fields and recorded the names, medical uses, and parts of the medicinal plants used.

Snowball sampling was used to identify 132 other informants, 66 males and 66 females. The methods of semistructured interviews and guided field walks and focal group discussions were adopted to collect information. The questions included the name, gender, nationality, age, family address, contact information, and other information of the informants, as well as whether they knew and used medicinal plants and the diseases that can be treated effectively.

Fresh and dried plant materials identified during interviews were collected as voucher specimens and deposited in the herbarium at the Minzu University of China in Beijing, China, for future reference. The botanical identities of voucher specimens were confirmed by the authors and other botanists at the Minzu University of China. Plant names were cross-checked with *Flora of China* (http://flora.huh.harvard.edu/china/) and digital resources from the internet, including http://www.tropicos.org/ and http://www.worldfloraonline.org.

### Data analysis

The data were cleaned and inputted into Microsoft Office Excel for statistical analysis. An ANOVA was used to compare the number of medicinal plants recognized by herbal doctors between age-groups and townships [34]. The relative frequency of citation (RFC) was used to evaluate the most common plant species used by local healers to treat various diseases, using the following formula:

$${\text{RFC}} = {\text{FC}}/{\text{N}}$$

where FC is the number of prescriptions mentioning the use of a plant species and *N* is the total number of prescriptions in this survey [35].

## Results and discussion

### Key and other informants

Information on a total of 15 local healers was collected through this study, which encompassed six townships. Healers started treating patients by setting up stalls at village fairs, inviting patients to their homes, and providing door-to-door medicinal services [36]. At the township level, Jiuqian township has five identified local healers, followed by Pu’an (3), Zhouqin (2), Dujiang (2), Dahe (2), and only one local healer in Zhonghe (Table 1). These local healers have little or no formal training or education. Their medicinal knowledge is mainly acquired through family inheritance, other healers, or self-study. The specialized Shui healers conduct their treatments in private clinics or township hospitals.

**Table 1:** Profiles of local Shui healers

Region	Records	Gender		Average age	Types of Shui doctors			Age of Shui doctors			Number of plants recognized
		Male	Female		I	II	III	< 40	40–60	> 60	
Jiuqian Township	5	5		44	3	1	1	1	3	1	213
Zhouqin Township	2	2		64	2				1	1	242
Dujiang Township	2	2		56	1	1			1	1	181
Dahe Township	2	2		53	1		1	1	1		205
Zhonghe Township	1		1	48	1				1		214
Pu’an Township	3	2	1	60	2		1		1	2	215
Total	15	13	2	54	10	2	3	2	8	5	
Number of plants recognized								127	204	236	

I—learnt from parents; II—learnt by self; III—learnt from teacher or master

Like other ethnic minorities [37–39], Shui communities also face challenges brought by Western medicine to preserve traditional medicine. This is because of modernization and urbanization that have resulted in fewer young people wanting to learn and practice traditional medicine. Low salary is also a fundamental issue that deters younger generations from learning traditional ethnomedicinal knowledge. As a result, the healer demographics skew older, an average age of 54, with only two healers under 40 years old. Healers were grouped by age, and a significant difference in the number of plants recognized by herbal doctors in the three age-groups (*F* = 54.870, *p* = 0.002, ANOVA) was identified (Table 2). The results show that older Shui healers have accumulated a rich experience in medicinal plant collection, identification, and treatments. However, ANOVA of the relationships among the number of medicinal plant species recognized in each township, found there was no difference in this study. This suggests that the traditional healers from different townships have a similar knowledge base of medicinal plants, and patients would likely get comparable therapy no matter in what township they sought traditional treatment (*F* = 0.341, *p* = 0.566, ANOVA) (Table 3).

**Table 2:** ANOVA of medicinal plants

Model		Sum of squares	df	Mean square	*F*	Sig.
1	Regression^a^	8333.333	1	8333.333	54.870	.002^b^
	Residual	607.500	4	151.875		
	Total	8940.833	5			

a. Dependent Variable: number of medicinal plants

b. Predictors: (Constant), age-group

**Table 3:** ANOVA of number of medicinal plants in townships

Model		Sum of squares	df	Mean square	*F*	Sig.
1	Regression^a^	4542.322	1	4542.322	.341	.566^b^
	Residual	239,717.878	18	13,317.660		
	Total	244,260.200	19			

a. Dependent Variable: number of plants

b. Predictors: (Constant), name of township

As for the gender structure of the healers, the Shui traditional healers included 13 male and 2 female healers. Most Shui traditional healers pass on their medical skills and knowledge to their sons, and in some cases, their nephews. This male progenitor transmission of traditional knowledge is a common form of inheritance throughout southwest China, such as Miao [40], Yao [41], and Dong [42]. However, this male progenitor system limits the pool of potential healers to only men directly related to the practitioner, and not others in the community, including women or unrelated individuals with an interest in health. Shui men traditionally collect plants, often in remote mountainous areas, and therefore learn more about the diverse medicinal flora of the region, whereas women are more likely to remain closer to home to attend domestic duties. This cultural norm further reinforces the male progenitor custom of healer selection.

Thus, to break this male progenitor system of medical knowledge inheritance, the Shui community is encouraged to collaborate with medical colleges and universities in Guizhou to establish a department of Shui ethnic medicine. This will enable a more diverse demographic of Shui people to receive systematic education and training.

As shown in Table 2, the *F* statistic is 54.870, and the sig value is 0.002 < 0.01, so there is a significant difference in the number of medicinal plants recognized by healers in the three age-groups.

As shown in Table 3, the degree of freedom is 1, the *F* value is 0.341, and the sig value is 0.566 > 0.01, so there is no significant difference in the number of medicinal plants recognized in each township.

A total of 132 informants from different demographic groups were interviewed on the therapeutic properties of medicinal plants throughout the six townships under study. As indicated in Table 4, informants were equally divided between females and males. They constituted five age-groups falling in the range of 18–92 years, with the majority being 20–79 years. Nine young people under the age of 20 were reluctant to use herbs, and only two thought herb use was “great,” 21 people between the ages of 20 and 39 were reluctant to use herbs, and six thought herb use was “great,” while more people in their 40 s and older think of using herbal medicine first when they get sick. Moreover, people over 60 are the most satisfied with the efficacy of herbal medicine.

**Table 4:** Informant demographic data and ethnobotanical data

Age-group	< 20 years of age												Total	Are the common diseases used first?		How have they worked?		
Township	Jiuqian		Dujiang		Zhonghe		Pu’an		Dahe		Zhouqin		12	Y	N	Bad	Good	Great
Gender	M	F	M	F	M	F	M	F	M	F	M	F		3	9	4	6	2
	1	1	1	1	1	1	1	1	1	1	1	1						

Age-group	20–39 years of age												Total	Are the common diseases used first?		How have they worked?		
Township	Jiuqian		Dujiang		Zhonghe		Pu’an		Dahe		Zhouqin		36	Y	N	Bad	Good	Great
Gender	M	F	M	F	M	F	M	F	M	F	M	F		15	21	5	25	6
	3	3	3	3	3	3	3	3	3	3	3	3						

Age-group	40–59 years of age												Total	Are the common diseases used first?		How have they worked?		
Township	Jiuqian		Dujiang		Zhonghe		Pu’an		Dahe		Zhouqin		36	Y	N	Bad	Good	Great
Gender	M	F	M	F	M	F	M	F	M	F	M	F		19	17	3	27	6
	3	3	3	3	3	3	3	3	3	3	3	3						

Age-group	60–79 years of age												Total	Are the common diseases used first?		How have they worked?		
Township	Jiuqian		Dujiang		Zhonghe		Pu’an		Dahe		Zhouqin		36	Y	N	Bad	Good	Great
Gender	M	F	M	F	M	F	M	F	M	F	M	F		27	9	3	24	9
	3	3	3	3	3	3	3	3	3	3	3	3						

Age-group	> 80 years of age												Total	Are the common diseases used first?		How have they worked?		
Township	Jiuqian		Dujiang		Zhonghe		Pu’an		Dahe		Zhouqin		12	Y	N	Bad	Good	Great
Gender	M	F	M	F	M	F	M	F	M	F	M	F		11	1	0	5	6
	1	1	1	1	1	1	1	1	1	1	1	1						

The results presented in Table 4 suggest that the demographic variables of informants influence the traditional medicinal plant knowledge of use and efficacy. Data on age-groups clearly show that older people rely more on medicinal plants. Many older people have gained in-depth knowledge of traditional medicine not only from their ancestors, but from their own observations over many years of utilization. Nevertheless, the lack of knowledge among younger generations may be due to changing lifestyles and waning interest in traditional medicine. These changes indicate a high risk of losing such important cultural heritage, and ways to prevent this loss are urgently needed.

### Diversity of medicinal plants within the study area

**Fig. 3:**
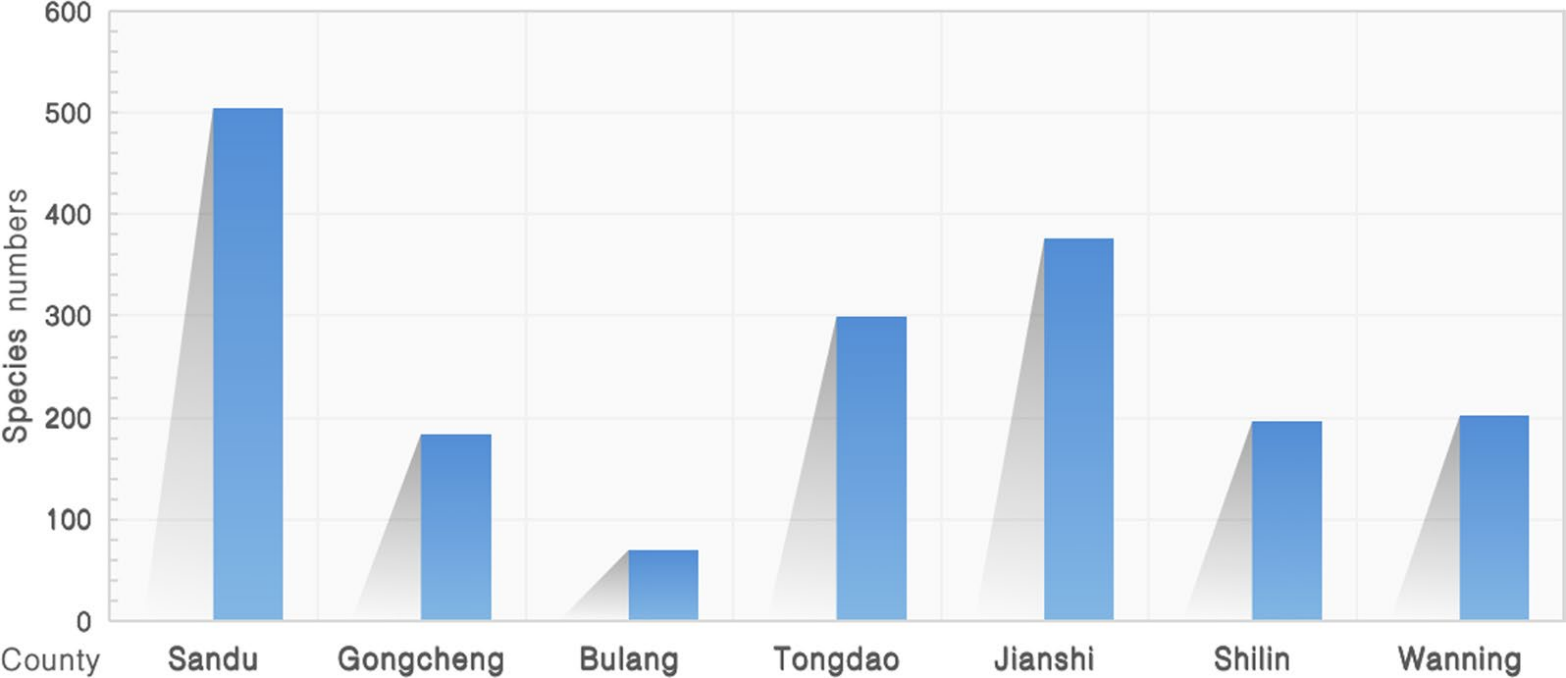
Comparison of species number of medicinal plants used in Sandu and other counties in China

A total of 505 species in 405 genera and 156 families of medicinal plants were documented in the Shui communities (Table 5). Among these 505 species, 130 species were identified for the first time as having medicinal usage, which is indicated by an asterisk in Table 5. The results also provided information on each species, including scientific name, family, Chinese name, local name, distribution, part used, use and value, preparation method, and voucher specimen number (Table 5). Compared with ethnomedicinal documentation in other communities, such as Yao people in Gongcheng County [41], Li people in Wanning [43], Bulang people in Menghai County [44], Tujia and Miao people in Jianshi County [45], Dong people in Tongdao [46] and Yi people in Shilin [47], the Shui medicinal species are especially diverse (Fig. 3). Many of the Shui medicinal plants have the typical characteristics of subtropical species. For example, herbs are mostly annual, and shrubs have strong adaptability and fast growth.

**Table 5:** Inventory of 505 medicinal plants used by Shui people in Sandu County

Scientific name	Family	Shui name	Chinese name (pin yin)	Part used	Habit	Distribution	Use and value	Preparation method	Voucher number	Remarks
*Abutilon theophrasti* Medicus	Malvaceae	Gan mei	Qing ma	Whole plant	Herb	Dahe; Sanhe	Heat-clearing and detoxifying; lactagogue; dysentery	Grinding, decoction; orally soup; pound fresh part applied on the affected area	SD-083	
*Acalypha australis* L.	Euphorbiaceae	Ma guang peng	Tie xian cai	Whole plant	Herb	Whole county	Dysentery; antitussive	Orally soup; pound fresh part applied on the affected area	SD-212	
*Acalypha supera* Forsskal	Euphorbiaceae	Ma gong bao	Ji yan cao	Whole plant	Herb	Puan; Shuilong	Dysentery	Orally soup; pound fresh part applied on the affected area	SD-203	
*Achillea millefolium* L.	Asteraceae	Ma ka ba	Shi	Whole plant	Herb	Dujiang; Jiuqian	Bruises; relieving rheumatism and cold; remove coldness	Grinding, decoction	SD-231	
*Achyranthes aspera* L.	Amaranthaceae	Ma du lve han	Liu ye niu xi	Root	Herb	Whole county	Bruises; the blood circulation hematischesis; heat-clearing and detoxifying	Grinding, decoction	SD-188	
*Achyranthes bidentata* Bl.	Amaranthaceae	Ma du lei wu	Niu xi	Root	Herb	Whole county	Blood circulation	Grinding and drink with wine	SD-187	
*Aconitum carmichaelii* Debeaux	Ranunculaceae	Ma gu luo	Wu tou	Tuber	Herb	Whole county	Dispelling wind and eliminating dampness	Grinding, decoction	SD-210	
*Acorus calamus* L.	Acoraceae	Xing fu ga	Chang pu	Rhizome	Herb	Whole county	Harmonizing stomach; relieving rheumatism and cold	Grinding, decoction	SD-455	
*Acorus gramineus* Soland	Acoraceae	Qian pu	Jin qian pu	Whole plant	Herb	Dahe; Sanhe; Dujiang	Heat-clearing and detoxifying; bruises	Grinding, decoction	SD-393	*
*Acorus tatarinowii* Schott	Acoraceae	Xing fu ga	Shi chang pu	Rhizome	Herb	Whole county	Spleen strengthen; Promoting eruption and promoting spleen yang	Orally soup	SD-456	
*Actinidia chinensis* Planch	Actinidiaceae	Yao nuan	Mi hou tao	Fruit; root	Shrub	Dujiang; Sanhe	Removing stasis; invigorates the spleen and promotes digestion	Decoction	SD-485	
*Actinidia rubricaulis* Dunn	Actinidiaceae	Fang man di	Mao hua yang tao	Root; leaf	Shrub	Dujiang; Sanhe	Heat-clearing and detoxifying; gastric cancer	Grinding, decoction	SD-079	
*Adenophora petiolata* subsp. *hunanensis* (Nannfeldt) D. Y. Hong & S. Ge	Campanulaceae	Ding dian hai	Xing ye sha shen	Root	Herb	Dujiang; Dahe	Eliminating phlegm and stopping cough	Orally soup	SD-054	
*Adiantum capillus-veneris* L.	Pteridaceae	Yao lan man	Tie xian jue	Whole plant	Herb	Dujiang	Dispelling wind and eliminating dampness	Grinding, decoction	SD-478	
*Aeginetia indica* L.	Orobanchaceae	Tu ling zhi cao	Ye gu	Whole plant	Herb	Dujiang; Zhouqin	Inflammation	Decoction	SD-428	*
*Agrimonia pilosa* Ldb.	Rosaceae	Ma ban bie	Long ya cao	Whole plant; root	Herb	Whole county	Dysentery; stop bleeding	Grinding, decoction	SD-166	
*Ailanthus altissima* (Mill.) Swingle	Simaroubaceae	Ju hai	Chou chun	Bark; fruit	Tree	Whole county	Dispelling wind and eliminating dampness; removing stasis	Medicated bath	SD-146	
*Ainsliaea fragrans* Champ.	Asteraceae	Pa zheng	Xing xiang tu er feng	Whole plant	Herb	Jiuqian	Bruises; heat-clearing and detoxifying	Fresh herbs are placed on the affected area	SD-385	
*Akebia trifoliata* (Thunb.) Koidz	Lardizabalaceae	Yao bing	Bai mu tong	Stem	Shrub	Dujiang; Jiuqian	Dispelling wind and eliminating dampness	Grinding, decoction	SD-465	
*Alangium chinense* (Lour.) Harms	Cornaceae	Mei an	Ba jiao feng	Root; leaf; flower	Tree	Whole county	Expelling wind-damp; bruises	Grinding, decoction	SD-300	
*Albizia julibrissin* Durazz.	Fabaceae	Mei ka	He huan	Bark	Tree	Whole county	Regulating qi; bruises	External application	SD-343	
*Aletris spicata* (Thunb.) Franch	Nartheciaceae	Jin xian diao bai mi	Fen tiao er cai	Whole plant	Herb	Whole county	Dysentery; ascaris	Grinding, decoction	SD-144	*
*Aleuritopteris anceps* (Blanford) Panigrahi	Pteridaceae	Jia fen bei jue	Fen bei jue	Whole plant	Herb	Dujiang	Dysentery	Grinding, decoction; orally soup	SD-139	*
*Aleuritopteris argentea* (Gmél.) Fée	Adiantaceae	Tong jing cao	Yin fen bei jue	Whole plant	Herb	Dujiang	The blood circulation hematischesis	Grinding, decoction; orally soup	SD-424	*
*Allium macrostemon* Bunge	Amaryllidaceae	Ye xie	Xie bai	Stem	Herb	Whole county	Dysentery	Grinding, decoction	SD-491	*
*Alpinia oblongifolia* Hayata	Zingiberaceae	Xing di duan	Hua shan jiang	Whole plant; root	Herb	Dujiang; Dahe	Harmonizing stomach; eliminating cold stop pain	Grinding, decoction	SD-454	
*Amana edulis* (Miq.) Honda	Liliaceae	Guang ci gu	Lao ya ban	Stem	Herb	Whole county	Moistening lung for arresting cough	Grinding, decoction	SD-113	*
*Amaranthus spinosus* L.	Amaranthaceae	Ma gu ga dian	Ci xian	Whole plant; root	Herb	Whole county	Venomous snake bite; heat-clearing and detoxifying	Fresh herbs are placed on the affected area	SD-209	
*Ampelopsis glandulosa* (Wall.) Momiy.	Vitaceae	Yin hui	She pu tao	Rhizome	Fungi	Dujiang; Sanhe	Dispelling wind and eliminating dampness; stop bleeding	Fresh herbs are placed on the affected area	SD-492	
*Androsace umbellata* (Lour.) Merr.	Primulaceae	Ma ge	Dian di mei	Whole plant; fruit	Herb	Whole county	Strong bones and muscles	Orally soup; pound fresh part applied on the affected area	SD-198	
*Anemone rivularis* Buch.-Ham.	Ranunculaceae	Du ding	Cao yu mei	Whole plant; root	Herb	Shuilong; Zhouqin	Removing stasis	Fresh herbs are placed on the affected area	SD-065	
*Anthriscus sylvestris* (L.) Hoffm.	Apiaceae	Tian qi	E shen	Root	Herb	Whole county	Nourishing liver and kidney; tranquilization	Orally soup	SD-421	*
*Ardisia crispa* (Thunb.) A. DC.	Primulaceae	Ba zhua long	Bai liang jin	Whole plant	Shrub	Dujiang	Eliminating phlegm and stopping cough	Grinding, decoction; orally soup	SD-013	*
*Ardisia densilepidotula* Merr.	Primulaceae	Ha mu lai	Mi lin zi jin niu	Root; leaf	Tree	Whole county	Expelling wind-damp	Grinding, decoction	SD-122	
*Ardisia japonica* (Thunberg) Blume	Primulaceae	Za du	Zi jin niu	Stem; root	Shrub	Dujiang; Dahe	Hemostasis; bruises	Grinding, decoction	SD-498	
*Argentina lineata* (Trevir.) Soják	Rosaceae	Ma jie ren man	Xi nan jue ma	Whole plant; root	Herb	Whole county	Gastroenteritis	Decoction	SD-229	
*Arisaema heterophyllum* Blume	Araceae	Ma da wan	Tian nan xing	Tuber	Herb	Dujiang; Dahe	Relieving dryness and moistening; eliminating phlegm and stopping cough	Pound fresh part applied on the affected area	SD-172	
*Aristolochia debilis* Sieb. et Zucc.	Aristolochiaceae	Ha du	Ma dou ling	Fruit; stem; root	Fungi	Dujiang; Jiuqian	Moistening lung for suppressing cough	Orally soup	SD-116	
*Aristolochia tubiflora* Dunn	Aristolochiaceae	Yao man long	Guan hua ma dou ling	Root; fruit	Herb	Dujiang; Jiuqian	Moistening lung for suppressing cough	Grinding, decoction	SD-483	*
*Artemisia argyi* Lévl. et Van	Asteraceae	Wa ai	Ai	Leaf; fruit	Herb	Dahe; Sanhe	Tocolysis; regulating the menstrual function to stop pain	Medicated bath	SD-430	
*Arthraxon hispidus* (Trin.) Makino	Poaceae	Lu zhu	Jin cao	Leaf; stem	Herb	Whole county	Eliminating phlegm and stopping cough	Grinding, decoction; orally soup	SD-159	*
*Asarum forbesii* Maxim.	Aristolochiaceae	Huai	Du heng	Whole plant	Herb	Dujiang; Jiuqian	Moistening lung for suppressing cough	Grinding, decoction; orally soup	SD-133	*
*Asarum insigne* Diels	Aristolochiaceae	Ma guang wa	Jin er huan	Whole plant	Herb	Whole county	Cough and expectorant, blood stasis and swelling	Grinding, external	SD-225	
*Asarum macranthum* Hook. f.	Aristolochiaceae	Ma bu hui	Da hua xi xin	Root	Herb	Dujiang; Jiuqian	Relieving rheumatism and cold; eliminating cold stop pain	Grinding, decoction	SD-170	
*Asarum sieboldii* Miq.	Aristolochiaceae	Ma guang wa	Han Cheng xi xin	Whole plant	Herb	Dujiang; Jiuqian	Dispelling wind and eliminating dampness	Grinding, decoction	SD-213	
*Asparagus cochinchinensis* (Lour.) Merr.	Asparagaceae	Ba bai zai	Tian men dong	Tuber	Herb	Whole county	Moistening lung for arresting cough	Boiled with meat and drunk the soup	SD-007	
*Aspidistra elatior* Blume	Asparagaceae	Ya ga	Zhi zhu bao dan	Rhizome	Herb	Dujiang; Sanhe	Bruises; decreasing swelling to relieving pain	Decoction	SD-460	
*Asplenium pekinense* Hance	Aspleniaceae	Da fei cao	Bei jing tie jiao jue	Whole plant	Herb	Dujiang	Eliminating phlegm and stopping cough	Grinding, decoction; orally soup	SD-040	*
*Asplenium trichomanes* L.Sp.	Aspleniaceae	Gang du gun	Tie jiao jue	Whole plant	Herb	Sanhe	Drainage of pus and dissolving carbuncle	Grinding, decoction	SD-089	
*Asplenium unilaterale* Lam.	Aspleniaceae	Dan bian tie jiao jue	Ban bian tie jiao jue	Whole plant	Herb	Dujiang	Infantile convulsion	Grinding, decoction; orally soup	SD-045	*
*Aster indicus* L.	Asteraceae	Ma da wan	Ma lan	Whole plant; root	Herb	Whole county	Heat-clearing and detoxifying; clearing heat; dehumidification	Grinding, decoction; orally soup	SD-173	
*Bassia scoparia* (L.) A.J. Scott	Amaranthaceae	Bai ni fan	Di fu	Whole plant	Herb	Whole county	Heat-clearing and detoxifying; clearing heat; dehumidification	Decoction	SD-016	
*Begonia grandis* Dry.	Begoniaceae	Ba lao ling	Qiu hai tang	Whole plant	Herb	Dujiang; Jiuqian	Dysentery; stanch flooding	Fresh herbs are placed on the affected area	SD-011	
*Belamcanda chinensis* (L.) Redouté	Iridaceae	Ma you	She gan	Rhizome	Herb	Whole county	Eliminating phlegm and stopping cough	Grinding, decoction	SD-287	
*Benincasa hispida* (Thunb.) Cogn.	Cucurbitaceae	Bai gua	Dong gua	Fruit; seed	Herb	Whole county	Inflammation	Boiled with meat and drunk the soup	SD-015	*
*Berberis sargentiana* Schneid.	Berberidaceae	Mei du ma	Ci hei zhu	Rhizome	Shrub	Dujiang; Jiuqian; Puan	Relieving exterior syndrome; heat-clearing and detoxifying	Fresh herbs are placed on the affected area	SD-321	*
*Berchemia lineata* (L.) DC.	Rhamnaceae	Sheng du	Tie bao jin	Root; leaf	Fungi	Whole county	Moistening lung for arresting cough	Orally soup; pound fresh part applied on the affected area	SD-408	
*Bergenia purpurascens* (Hook. f. et Thoms.) Engl.	Saxifragaceae	Ma ba ding	Yan bai cai	Whole plant	Herb	Whole county	Nourishing yin; strangury	Orally soup; pound fresh part applied on the affected area	SD-163	
*Bidens bipinnata* L.	Asteraceae	Du ding	Po po zhen	Whole plant	Herb	Puan	Dysentery; gastroenteritis	Decoction	SD-066	
*Bistorta paleacea* (Wall. ex Hook. f.) Yonekura et H. Ohashi	Polygonaceae	Gang zhan lu	Cao xue jie	Rhizome	Herb	Dahe; Puan	Eliminating phlegm and stopping cough	Decoction	SD-102	
*Bletilla striata* (Thunb. ex Murray) Rchb. f.	Orchidaceae	Gang jie ba	Bai ji	Bulbs	Herb	Whole county	Spleen strengthen	Grinding	SD-095	
*Boehmeria penduliflora* Wedd. ex D.G. Long	Urticaceae	Mei ha na	Zhang ye zhu ma	branch; root	Shrub	Whole county	Dispelling wind and eliminating dampness	Decoction	SD-338	
*Brandisia hancei* Hook. f.	Orobanchaceae	Ma miao	Lai jiang teng	Whole plant	Shrub	Puan; Zhouqin	Dysentery; dispelling wind and eliminating dampness	Grinding, decoction	SD-252	
*Broussonetia papyrifera* (L.) L'Hér. ex Vent.	Moraceae	Mei ha	Gou	Fruit	Tree	Whole county	Tonifying kidney	Orally soup	SD-337	
*Burmannia coelestis* D. Don	Burmanniaceae	Qiu nu jia	San pin yi zhi hua	Rhizome	Herb	Whole county	Promoting eruption and promoting spleen yang	Decoction	SD-398	
*Buxus sinica* (Rehder & E. H. Wilson) M. Cheng	Buxaceae	Mei mao	Huang yang	Root; leaf	Shrub	Shuilong; Zhouqin	Eliminating phlegm and stopping cough	Medicinal liquor; pound fresh part applied on the affected area	SD-350	
*Calanthe discolor* Lindl.	Orchidaceae	Wa jiu qiu	Xia ji lan	Root	Herb	Whole county	Heat-clearing and detoxifying	Grinding, decoction	SD-433	
*Callerya dielsiana* (Harms) P. K. Loc ex Z. Wei & Pedley	Fabaceae	Yao nai	Xiang hua ji xue teng	Root	Fungi	Dujiang; Zhouqin	Expelling wind-damp	Grinding, decoction; orally soup	SD-484	
*Callicarpa macrophylla* Vahl.	Lamiaceae	Mei lou lu	Da ye zi zhu	Leaf; root	Tree	Whole county	Bruises; stop bleeding	Fresh herbs are placed on the affected area	SD-347	
*Calocedrus macrolepis* Kurz.	Cupressaceae	Nv mei ou	Cui bai	Fruit	Tree	Whole county	Expelling wind-damp	Grinding, decoction	SD-382	
*Calystegia hederacea* Wall.	Convolvulaceae	Ma xiang han	Da wan hua	Whole plant; root	Herb	Whole county	Regulate the menstrual function to stop pain	Fresh herbs are placed on the affected area	SD-278	
*Camellia sinensis* (L.) O. Ktze.	Theaceae	Mei za	Cha	Leaf; root	Tree	Whole county	Cardiotonic	Decoction	SD-364	
*Campanumoea javanica* Bl.	Campanulaceae	Nai shen	Jin qian bao	Root	Fungi	Whole county	Strengthen the spleen; harmonizing stomach	Boiled with meat and drunk the soup	SD-371	*
*Campsis grandiflora* (Thunb.) Schum.	Bignoniaceae	Dou du gun	Ling xiao	Flower; rhizome	Fungi	Dujiang; Dahe	Fracture	Orally soup	SD-063	
*Campylotropis hirtella* (Franch.) Schindl.	Fabaceae	Ma jie suo	Mao guang zi shao	Whole plant	Shrub	Dahe; Zhouqin	Removing stasis; regulate the menstrual function to stop pain	Grinding, decoction	SD-230	
*Capsella bursa-pastoris* (L.) Medic.	Brassicaceae	Ma ding jie	Ji	Whole plant	Herb	Whole county	Improving eyesight and removing nebula; anticancer	Boiled with meat and drunk the soup	SD-180	
*Caragana sinica* (Buchoz) Rehd.	Fabaceae	Nv yue di	Jin ji er	Flower; root	Shrub	Dujiang; Sanhe	Moistening lung for arresting cough	Decoction	SD-384	
*Cardamine lyrata* Bunge	Brassicaceae	A jiu ying	Shui tian sui mi ji	Leaf; stem	Herb	Whole county	Dispelling wind and eliminating dampness	Grinding, decoction; orally soup	SD-001	*
*Cardiocrinum giganteum* (Wall.) Makino	Liliaceae	Qiu an	Da bai he	Bulbs; flower; seed	Herb	Dujiang; Shuilong	Decreasing swelling to relieving pain; inducing diuresis for removing edema	Fresh herbs are placed on the affected area	SD-396	
*Carpesium cernuum* L.	Asteraceae	Yan dai cao	Yan guan tou cao	Whole plant	Herb	Whole county	Venomous snake bite	Fresh herbs are placed on the affected area	SD-462	*
*Castanea mollissima* Blume	Fagaceae	Dai	Li	Root	Tree	Whole county	Inducing diuresis for removing edema	Orally soup	SD-044	
*Castanopsis tibetana* Hance	Fagaceae	Mei dai	Gou zhui	Fruit	Tree	Whole county	Gastroenteritis	Grinding, decoction; orally soup	SD-312	
*Catalpa bungei* C. A. Mey	Bignoniaceae	Wang mu	Qiu	Seed	Tree	Dahe; Sanhe	Drainage of pus and dissolving carbuncle	Fresh herbs are placed on the affected area	SD-438	*
*Catalpa ovata* G. Don.	Bignoniaceae	Mei duo na	Zi	Root; leaf; fruit	Tree	Dahe; Sanhe	Promoting blood circulation; promoting flow of qi and blood circulation	Grinding, decoction	SD-324	
*Causonis japonica* (Thunb.) Raf.	Vitaceae	Yin ya lao	Wu lian mei	Whole plant; root	Fungi	Whole county	Expelling wind-damp	Grinding, decoction	SD-495	
*Celastrus orbiculatus* Thunb.	Celastraceae	Nan she feng	Nan she teng	Rhizome; leaf; fruit	Shrub	Dahe; Dujiang	Blood circulation	Grinding, decoction; orally soup	SD-373	*
*Celosia argentea* L.	Amaranthaceae	Gang gen gu	Qing xiang	Seed	Herb	Dahe; Sanhe	Improving eyesight and removing nebula	Decoction	SD-092	
*Celosia cristata* L.	Amaranthaceae	Ma wu ga han	Ji guan hua	Flower	Herb	Dahe; Sanhe	Dysentery; hemorrhoids	Grinding, decoction	SD-275	
*Celtis sinensis* Pers.	Cannabaceae	Mei xiu di	Po shu	Bark	Tree	Whole county	Heat-clearing and detoxifying; regulating the menstrual function to stop pain	Grinding, decoction	SD-361	
*Centella asiatica* (L.) Urban	Apiaceae	Ma kui wa lao	Ji xue cao	Whole plant	Herb	Whole county	Removing stasis; inducing diuresis for removing edema	Fresh herbs are placed on the affected area	SD-239	
*Centipeda minima* (L.) A. Br. et Aschers.	Asteraceae	Ma jie gu	Shi hu sui	Whole plant	Herb	Whole county	Heat-clearing and detoxifying; relieving rheumatism and cold	Grinding and drink with wine	SD-227	
*Cephalotaxus fortunei* Hooker	Taxaceae	Mei fan meng	San jian shan	Seed	Tree	Dujiang; Jiuqian	Moistening lung for arresting cough	Stir fry	SD-327	
*Cephalotaxus oliveri* Mast.	Taxaceae	A li shan	Bi zi san jian shan	Leaf; seed	Shrub	Dujiang; Jiuqian	Analgesic and hemostasis	Decoction	SD-002	*
*Cerastium fontanum* subsp. *vulgare* (Hartman) Greuter & Burdet	Caryophyllaceae	Xia ye quan juan er	Cu sheng quan juan er	Whole plant	Herb	Whole county	Heat-clearing and detoxifying	Grinding, decoction	SD-444	*
*Ceratophyllum demersum* L.	Ceratophyllaceae	Xi cao	Jin yu zao	Whole plant	Herb	Whole county	Hemostasis; stanch flooding	Decoction	SD-442	*
*Cercis chinensis* Bunge	Fabaceae	Zi zhu	Zi jing	Bark; root	Tree	Dahe; Sanhe	Blood circulation	Grinding, decoction	SD-505	*
*Chamaecrista leschenaultiana* (Candolle) O. Degener	Fabaceae	Di you gan	Duan ye shan bian dou	Root; leaf; root	Herb	Whole county	Gastroenteritis; harmonizing stomach	Grinding, decoction; orally soup	SD-053	*
*Cheniella glauca* (Benth.) R. Clark & Mackinder	Fabaceae	Mei wa e	Fen ye shou guan teng	Root	Tree	Whole county	Strengthen waist and sinews	Medicinal liquor	SD-356	*
*Chenopodium album* L.	Amaranthaceae	Ma mei feng	Li	Whole plant	Herb	Whole county	Dysentery; harmonizing stomach	Decoction	SD-248	
*Chloranthus holostegius* (Hand.-Mazz.) C.Pei & San	Chloranthaceae	Cha ye lan	Quan yuan jin su lan	Whole plant	Herb	Dahe; Dujiang	Antibacterial insecticide	Grinding, decoction	SD-032	*
*Chlorophytum comosum* (Thunb.) Baker	Asparagaceae	Gang yan nuo	Diao lan	Whole plant; root	Herb	Whole county	Moistening lung for arresting cough	Decoction; decoction	SD-098	
*Choerospondias axillaris* (Roxb.) B. L. Burtt & A. W. Hill	Anacardiaceae	Wu yan guo	Nan suan zao	Bark; fruit	Tree	Dujiang	Empyrosis	Grinding, decoction; orally soup	SD-441	*
*Chrysanthemum indicum* Linnaeus	Asteraceae	Ku yi	Ye ju	Flower	Herb	Whole county	Improving eyesight and removing nebula	Grinding, decoction	SD-148	*
*Cibotium barometz* (L.) J. Sm.	Cibotiaceae	Yao ge man	Jin mao gou	Rhizome	Herb	Whole county	Nourishing liver and kidney; heat-clearing and detoxifying	Grinding, decoction	SD-473	
*Cinnamomum camphora* (L.) Presl.	Lauraceae	Mei ge lun	Zhang	Branch; root	Tree	Whole county	Centipede bites	Decoction	SD-335	
*Cinnamomum cassia* Presl.	Lauraceae	Yu gui	Rou gui	Stem	Tree	Dahe; Dujiang; Jiuqian	Harmonizing stomach	Grinding, decoction; orally soup	SD-497	*
*Cinnamomum parthenoxylon* (Jack) Meisner	Lauraceae	Mei dong	Huang zhang	Root	Tree	Whole county	Relaxing tendon and activation collaterals; remove coldness	Grinding, decoction; orally soup	SD-319	
*Cinnamomum wilsonii* Gamble	Lauraceae	Mei gei di	Chuan gui	Bark	Tree	Dujiang; Jiuqian	Relieving rheumatism and cold	Grinding, decoction	SD-336	
*Cirsium eriophoroides* (Hook. f.) Petrak	Asteraceae	Ma zai lao	Mian tou ji	Whole plant	Herb	Whole county	Removing stasis; hemostasis	Orally soup	SD-292	
*Citrus reticulata* Blanco	Rutaceae	Ju	Gan ju	Fruit	Tree	Whole county	Blood circulation	Decoction	SD-145	*
*Citrus sinensis* (L.) Osbeck cv. Xue Cheng	Rutaceae	Liu cheng	Tian cheng	Fruit	Tree	Whole county	Eliminating phlegm and stopping cough	Decoction	SD-156	*
*Clematis armandii* Franch.	Ranunculaceae	Yao lei	Xiao mu tong	Stem	Fungi	Whole county	Inducing diuresis for removing edema; apocenosis	Grinding, decoction	SD-480	
*Clematis chinensis* Osbeck	Ranunculaceae	Yao ji zu	Wei ling xian	Root	Fungi	Whole county	Expelling wind-damp	Grinding, decoction	SD-477	
*Clerodendrum bungei* Steud.	Lamiaceae	Ma kun han	Chou mu dan	Stem; leaf; root	Shrub	Whole county	Dispelling wind and eliminating dampness	Grinding, decoction	SD-240	
*Clinopodium chinense* (Benth.) O. Ktze.	Lamiaceae	Feng lun cai	Feng lun cao	Whole plant	Herb	Whole county	Promoting eruption and promoting spleen yang	Grinding, decoction	SD-080	*
*Clinopodium polycephalum* (Vaniot) C. Y. Wu et Hsuan ex P. S. Hsu	Lamiaceae	Ma ya ji	Deng long cao	Whole plant	Herb	Whole county	Clearing heat; anti-febrile; dehumidification	Grinding, decoction	SD-284	
*Cocculus orbiculatus* (L.) DC.	Menispermaceae	Yao ga lu	Mu fang ji	Root	Fungi	Dujiang; Jiuqian	Relieving rheumatism and cold; swelling and pain in throat	Orally soup; pound fresh part applied on the affected area	SD-471	
*Coix lacryma-jobi* L.	Hypericaceae	Ao bei	Yi yi	Seed	Herb	Whole county	Invigorating the spleen and promotes digestion	Orally soup	SD-005	
*Commelina communis* L.	Commelinaceae	Ma wa fan	Ya zhi cao	Whole plant	Herb	Whole county	Heat-clearing and detoxifying	A suitable amount used externally	SD-268	
*Coniogramme japonica* (Thunb.) Diels	Pteridaceae	Ma guai	Feng liao jue	Whole plant	Herb	Dahe	Inducing diuresis for removing edema	Medicinal liquor; orally soup	SD-211	
*Coriandrum sativum* L.	Apiaceae	Ma lao	Yan sui	Whole plant	Herb	Whole county	Indigestion	Grinding, decoction	SD-241	
*Coriaria nepalensis* Wall.	Coriariaceae	Mei shai	Ma sang	Leaf	Tree	Whole county	Empyrosis	Medicated bath	SD-353	
*Crataegus cuneata* Sieb. et Zucc.	Rosaceae	Jie nu	Ye shan zha	Fruit	Tree	Puan	Appetizing digestion	Decoction	SD-142	
Crepidiastrum denticulatum (Houttuyn) Pak & KawanoStebb. ssp. *pubescens* Stenbb.	Asteraceae	Ma ling gan	Huang gua cai	Whole plant	Herb	Whole county	Clearing and activating the channels and collaterals	Decoction	SD-244	
*Crotalaria albida* Heyne ex Roth	Fabaceae	Gang ding gui	Xiang ling dou	Whole plant	Herb	Whole county	Heat-clearing and detoxifying; inducing diuresis for removing edema	Fresh herbs are placed on the affected area	SD-087	
*Cryptomeria japonica* (Thunb. ex L.f.) D. Don	Cupressaceae	Za wa lao	Ri ben Liu shan	Bark	Tree	Whole county	Dermatosis	Medicated bath	SD-499	
*Cunninghamia lanceolata* (Lamb.) Hook.	Cupressaceae	Mei ao	Shan mu	Bark; root; leaf	Tree	Whole county	Dispelling wind and eliminating dampness	Grinding, decoction	SD-301	
*Curculigo orchioides* Gaertn.	Hypoxidaceae	Gang yu	Xian mao	Rhizome	Herb	Whole county	Nourishing liver and kidney	Grinding and drink with wine	SD-100	
*Curcuma longa* L.	Zingiberaceae	Xing ma	Jiang huang	Rhizome	Herb	Dahe; Jiuqian; Sanhe	Dehumidification; regulating the menstrual function to stop pain	Decoction	SD-458	
*Cycas revoluta* Thunb.	Cycadaceae	Tie shu	Su tie	Root; leaf; seed	Tree	Dahe; Dujiang	Eliminating phlegm and stopping cough	Decoction	SD-423	*
*Cyperus rotundus* L.	Cyperaceae	Gang yu long	Xiang fu zi	Rhizome	Herb	Dahe; Sanhe	Regulating the menstrual function to stop pain	Medicated bath	SD-101	
*Cyrtomium fortunei* J. Sm.	Dryopteridaceae	Mian ma lin mao jue	Guan zhong	Rhizome	Herb	Whole county	Killing parasites to relieve itching	Medicinal liquor	SD-367	*
*Dalbergia hancei* Benth.	Fabaceae	Duo bi lao	Teng huang tan	Stem; root	Fungi	Dujiang; Dahe	Strong bones and muscles; regulate the menstrual function to stop pain	Grinding, decoction	SD-069	
*Daphne odora* Thunb.	Thymelaeaceae	Xue dong hua	Rui xiang	Whole plant	Shrub	Jiuqian; Zhouqin	Strong bones and muscles; fracture	Grinding, decoction	SD-459	*
*Daphniphyllum macropodum* Miq.	Daphniphyllaceae	Hua mei zhu	Jiao rang mu	Leaf; seed	Tree	Dahe; Dujiang; Sanhe	Drainage of pus and dissolving carbuncle; promoting eruption	Fresh herbs are placed on the affected area	SD-132	*
*Datura stramonium* L.	Solanaceae	Mei deng bo luo	Man tuo luo	Flower; seed; leaf	Herb	Whole county	Antitussive; dispelling wind and eliminating dampness	Grinding, decoction	SD-314	
*Davallia trichomanoides* Blume.	Davalliaceae	Man jiao ni	Gu cui bu	Rhizome	Herb	Whole county	Strong bones and muscles	Orally soup; medicinal liquor	SD-296	
*Dendrobium moniliforme* (L.) Sw.	Orchidaceae	Gang bing	Xi jing shi hu	Stem	Herb	Dujiang; Sanhe	Health and thirst	Grinding, decoction	SD-085	
*Dichondra micrantha* Urban	Convolvulaceae	Ma kui dian	Ma ti jin	Whole plant	Herb	Whole county	Dehumidification	Orally soup	SD-237	
*Dicranopteris pedata* (Houttuyn) Nakaike	Gleicheniaceae	Mang qi	Mang qi	Whole plant	Herb	Whole county	Inducing diuresis for removing edema	Grinding, decoction	SD-297	*
*Dioscorea cirrhosa* Lour.	Dioscoreaceae	Man di	Shu liang	Tuber	Fungi	Dujiang; Dahe; Jiuqian	Hemoptysis; bleeding from five sense organs or subcutaneous tissue	Decoction	SD-293	
*Dioscorea melanophyma* Prain et Burkill	Dioscoreaceae	Ni dou	Hei zhu ya shu liang	Tuber	Fungi	Dujiang; Dahe	Stanch flooding	Fresh herbs are placed on the affected area	SD-375	
*Dioscorea polystachya* Turczaninow	Dioscoreaceae	Man di	Shu yu	Tuber	Fungi	Whole county	Spleen and stomach strengthen	Grinding, decoction; orally soup	SD-294	
*Diospyros kaki* Thunb.	Ebenaceae	Shi	Shi	Fruit	Tree	Whole county	Clear liver fire; blood cooling and arresting	Grinding, decoction; orally soup	SD-409	
*Diphasiastrum complanatum* (L.) Holub	Lycopodiaceae	Yao man hai	Bian zhi shi song	Whole plant	Herb	Dujiang	Dehumidification	Grinding, decoction	SD-481	*
*Dipsacus asper Wallich* ex Candolle	Caprifoliaceae	Ma zai	Chuan xu duan	Root	Herb	Whole county	Strengthening bones and muscles; nourishing liver and kidney	Grinding, decoction; orally soup	SD-290	
*Disporum cantoniense* (Lour.) Merr.	Colchicaceae	Ma heng bo	Wan shou zhu	Rhizome	Herb	Dahe; Sanhe	Moistening lung for arresting cough	Grinding, decoction	SD-217	
*Draba nemorosa* L.	Brassicaceae	Ma xiang lan	Ting li	Seed	Herb	Puan; Sanhe	Eliminating phlegm and stopping cough	Decoction	SD-279	
*Drosera peltata* Smith	Droseraceae	Ma mi da	Mao gao cai	Whole plant	Herb	Whole county	Bruises	Fresh herbs are placed on the affected area	SD-251	
*Drynaria propinqua* (Wall. ex Mett.) J. Sm. ex Bedd.	Polypodiaceae	Jin lin hu jue	Shi lian jiang hu jue	Rhizome	Herb	Lalan	Bruises	Medicinal liquor	SD-143	*
*Duchesnea indica* (Andr.) Focke	Rosaceae	She pao cao	She mei	Whole plant	Herb	Whole county	Heat-clearing and detoxifying	Grinding, decoction; orally soup	SD-407	*
*Duhaldea cappa* (Buchanan-Hamilton ex D. Don) Pruski & Anderberg	Asteraceae	Ma pang da	Yang er ju	Whole plant; root	Shrub	Whole county	Regulating the menstrual function to stop pain; eliminating cold stop pain	Grinding, decoction	SD-262	
*Dysosma versipellis* (Hance) M. Cheng ex Ying	Berberidaceae	Mei wa ban	Ba jiao lian	Rhizome	Herb	Dujiang; Jiuqian	Eliminating phlegm and stopping cough	Medicinal liquor	SD-355	
*Dysphania ambrosioides* (Linnaeus) Mosyakin & Clemants	Amaranthaceae	Ma nian	Tu jing jie	Whole plant	Herb	Whole county	Appetizing digestion	Orally soup	SD-257	
*Eclipta prostrata* (L.) L.	Asteraceae	Ma han xia	Li chang	Whole plant	Herb	Whole county	Strengthening bones and muscles	Grinding, decoction	SD-215	
*Elaeagnus pungens* Thunb.	Elaeagnaceae	Mei du	Hu tui zi	Fruit; root; leaf	Shrub	Dujiang; Sanhe	Antitussive; eliminating phlegm and stopping cough	Medicinal liquor	SD-320	
*Elatostema umbellatum* (Siebold & Zucc.) Blume	Urticaceae	Ma ai na	Shang tian ti	Whole plant	Herb	Whole county	Clearing heat; dehumidification	Medicinal liquor	SD-160	
*Elephantopus scaber* L.	Asteraceae	Ma za niang	Di dan cao	Whole plant	Herb	Whole county	Detumescence; relieving rheumatism and cold	Decoction	SD-289	
*Eleutherococcus nodiflorus* (Dunn) S. Y. Hu	Araliaceae	Ma gou pa	Wu jia	Root; leaf	Shrub	Dujiang; Sanhe	Strengthening bones and muscles	Grinding and drink with wine	SD-207	
*Eleutherococcus senticosus* (Ruprecht & Maximowicz) Maximowicz	Araliaceae	Ma gou e wa	Ci wu jia	Root	Shrub	Dujiang; Jiuqian; Zhouqin	Expelling wind-damp; Strong bones and muscles	Medicinal liquor	SD-204	
*Elsholtzia ciliata* (Thunb.) Hyland.	Lamiaceae	Xiao xiang ru	Xiang ru	Whole plant	Herb	Jiuqian; Zhouqin	Gastroenteritis; harmonizing stomach	Grinding, decoction; orally soup	SD-452	*
*Emilia sonchifolia* (L.) DC.	Asteraceae	Ma dian	Yi dian hong	Whole plant	Herb	Whole county	Heat-clearing and detoxifying; removing pathogenic heat from the blood and toxic material from the body	Grinding, decoction; orally soup	SD-176	
*Epimedium brevicornu* Maxim.	Berberidaceae	Yao wa jiu	Yin yang huo	Whole plant	Herb	Dujiang; Shuilong	Strong bones and muscles; manage qi and activating blood	Grinding, decoction	SD-487	
*Epipremnum pinnatum* (L.) Engl.	Araceae	Qi lin wei	Qi lin ye	Whole plant	Herb	Dujiang; Shuilong; Zhouqin	Detumescence and promoting granulation	Grinding, decoction; orally soup	SD-392	*
*Equisetum arvense* L.	Equisetaceae	Suo ma di	Wen jing	Stem	Herb	Whole county	Eliminating phlegm and stopping cough	Orally soup	SD-420	
*Equisetum hyemale* L.	Equisetaceae	Suo ma	Mu zei	Stem	Herb	Whole county	Bloody flux	Grinding, decoction	SD-418	
*Equisetum ramosissimum* Desf.	Equisetaceae	Suo ma	Jie jie cao	Stem	Herb	Whole county	Improving eyesight and removing nebula	Decoction	SD-419	
*Erigeron breviscapus* (Vant.) Hand.-Mazz.	Asteraceae	Ma duo wan	Duan ting fei peng	Whole plant	Herb	Jiuqian; Puan; Zhouqin	Blood circulation	Grinding, decoction	SD-191	
*Eriobotrya japonica* (Thunb.) Lindl.	Rosaceae	Xi mei xia	Pi pa	Leaf; root	Tree	Whole county	Antitussive; relieving dryness and moistening	Orally soup	SD-443	
*Eriocaulon buergerianum* Koern.	Eriocaulaceae	Wa er duo cao	Gu jing cao	Whole plant	Herb	Dujiang; Sanhe	Improving eyesight and removing nebula	Grinding, decoction	SD-432	*
*Eucalyptus robusta* Smith	Myrtaceae	Mei gang an	An	Leaf	Tree	Whole county	Expelling wind-damp; enteritis	Grinding, decoction	SD-332	
*Eucommia ulmoides* Oliv.	Eucommiaceae	Mei bi du	Du zhong	Bark; leaf	Tree	Dujiang; Jiuqian; Dahe	Clearing liver to add yin; nourishing liver and kidney	Medicinal liquor	SD-306	
*Euonymus alatus* (Thunb.) Sieb.	Celastraceae	Mei ga	Wei mao	Bark	Shrub	Whole county	Inducing diuresis for removing edema	Grinding, decoction	SD-329	
*Euphorbia lathyris* L.	Euphorbiaceae	Ma bai ni	Xu sui zi	Seed	Herb	Whole county	Dysentery	Grinding, decoction	SD-165	
*Euphorbia sikkimensis Boiss*.	Euphorbiaceae	Ma wa liu	Huang bao da ji	Root; leaf	Herb	Dujiang; Dahe	Dysentery; drainage of pus and dissolving carbuncle	Fresh herbs are placed on the affected area	SD-271	
*Euscaphis japonica* (Thunb.) Dippel	Staphyleaceae	Mei da jie	Ye ya chun	Root; fruit	Tree	Dujiang	Dispelling wind and eliminating dampness	Grinding, decoction	SD-311	
*Ficus carica* L.	Moraceae	Deng po	Wu hua guo	Flower; root; leaf	Shrub	Whole county	Relieving dryness and moistening; moisten the dry digestive apparatus	Decoction	SD-047	
*Ficus microcarpa* L. f	Moraceae	Bi ba ga	Rong shu	Bark	Tree	Whole county	Heat-clearing and detoxifying	Decoction	SD-025	
*Ficus pandurata* Hance	Moraceae	Cha ye niu nai zi	Qin ye rong	Root	Shrub	Dujiang; Sanhe	Relaxing tendon and activation collaterals	Grinding, decoction; orally soup	SD-033	*
*Ficus pumila* L.	Moraceae	Weng ba	Bi li	Rhizome; leaf; fruit	Shrub	Whole county	Dispelling wind and eliminating dampness	Medicinal liquor	SD-440	
*Ficus simplicissima* Lour.	Moraceae	Mei ding man	Ji jian rong	Root	Shrub	Dujiang; Sanhe	Expelling wind-damp; Dispelling wind and eliminating dampness	Decoction	SD-318	
*Ficus tikoua* Bur.	Moraceae	Wa yao hong	Di guo	Root; leaf	Fungi	Whole county	Dispelling wind and eliminating dampness	Grinding, decoction	SD-434	
*Foeniculum vulgare* Mill.	Apiaceae	Ma ding ma	Hui xiang	Fruit	Herb	Whole county	Appetizing digestion	Medicinal liquor	SD-181	
*Fraxinus chinensis* Roxb	Oleaceae	Mei bei na	Bai la shu	Bark	Tree	Whole county	Regulate the menstrual function to stop pain	Grinding, decoction	SD-305	*
*Ganoderma sinense* Zhao, Xu et Zhang	Polyporaceae	Zi ling zhi	Zi zhi	Herb	Herb	Whole county	Nourishing liver and kidney; relieve uneasiness of mind and body tranquilization	Decoction	SD-504	*
*Garcinia multiflora* Champ. ex Benth.	Hypericaceae	Shan ju zi	Mu zhu zi	Root; fruit; bark	Tree	Dujiang	Detumescence	Fresh herbs are placed on the affected area	SD-405	*
*Gardenia jasminoides* Ellis	Rubiaceae	Mei le	Zhi zi	Fruit	Shrub	Whole county	Blood cooling and arresting; heat-clearing and detoxifying	Decoction	SD-345	
*Gastrodia elata* Bl.	Orchidaceae	Ya na	Tian ma	Tuber	Herb	Dujiang; Dahe	Regulating the menstrual function to stop pain; clearing and activating the channels and collaterals	Grinding, decoction; orally soup	SD-461	
*Gaultheria leucocarpa* Bl.	Ericaceae	Shai nuo	Bai guo bai zhu	Stem; leaf; root	Shrub	Dujiang; Dahe	Dispelling wind and eliminating dampness	Grinding, decoction	SD-401	
*Gentiana scabra* Bunge	Gentianaceae	Gang duo ga	Long dan	Root	Herb	Dujiang; Jiuqian; Zhouqin	Eliminating phlegm and stopping cough; remove coldness	Grinding, decoction	SD-090	
*Geranium wilfordii* Maxim.	Geraniaceae	Ma xian gong	Lao guan cao	Whole plant	Herb	Whole county	Strong bones and muscles; relaxing tendon and activation collaterals	Orally soup	SD-277	
*Gerbera piloselloides* (L.) Cass.	Asteraceae	Ba hao	Tu er yi zhi jian	Whole plant	Herb	Whole county	Eliminating phlegm and stopping cough; moistening lung for arresting cough	Decoction	SD-010	
*Geum japonicum* Thunb.	Rosaceae	Ma kang	Ri ben lu bian qing	Whole plant	Shrub	Whole county	Decreasing swelling to relieving pain	Decoction	SD-235	
*Ginkgo biloba* L.	Ginkgoaceae	Mei ding a	Yin xing	Seed	Tree	Dujiang; Sanhe; Shuilong	Tonifying kidney; nourishing yang	Grinding, decoction	SD-315	
*Glandularia tenera* (Spreng.) Cabrera	Verbenaceae	Yan wei cao	Xi ye mei nv ying	Whole plant	Herb	Whole county	Blood circulation	Grinding, decoction; orally soup	SD-463	*
*Glechoma longituba* (Nakai) Kupr.	Lamiaceae	Ji hong lao	Huo xue dan	Whole plant; root	Herb	Dujiang; Jiuqian	Regulate the menstrual function to stop pain	Grinding, decoction; orally soup	SD-137	
*Gleditsia sinensis* Lam.	Fabaceae	Mei zao jiao	Zao jia	Fruit; root	Tree	Dujiang; Sanhe	Removing stasis	Decoction	SD-365	
*Glochidion puberum* (L.) Hutch.	Phyllanthaceae	Mei nv ban	Suan pan zi	Fruit	Shrub	Dujiang; Dahe	Antidiarrheic	Decoction	SD-352	
*Glycine soja* Siebold & Zucc.	Fabaceae	Lu dou	Ye da dou	Seed	Herb	Dahe; Dujiang	Inducing diuresis for removing edema	Grinding, decoction; orally soup	SD-157	*
*Goniophlebium niponicum* (Mett.) Yea C.Liu, W.L.Chiou & M.Kato	Polypodiaceae	La ga xiang	Ri ben shuilong gu	Rhizome	Herb	Dujiang	Bruises	Grinding, decoction	SD-151	
*Gonocarpus micranthus* Thunberg	Haloragaceae	Chuan ban cao	Xiao er xian cao	Whole plant	Herb	Whole county	Venomous snake bite, relieve swelling and pain	Fresh herbs are placed on the affected area	SD-036	*
*Gonostegia hirta* (Bl.) Miq.	Urticaceae	Ma ao xing	Nuo mi tuan	Whole plant	Herb	Whole county	Heat-clearing and detoxifying; clearing heat; dehumidification	Grinding, decoction; orally soup	SD-162	
*Goodyera prainii* Hook. f.	Orchidaceae	Wa dong gai	Chui ye ban ye lan	Whole plant	Herb	Dujiang; Shuilong	Enuresis	Grinding, decoction	SD-431	
*Goodyera schlechtendaliana* Rchb. f.	Orchidaceae	Ma bao dong	Ban ye lan	Whole plant	Herb	Dujiang; Sanhe	Heat-clearing and detoxifying; regulate qi	Grinding, decoction	SD-167	
*Gynostemma pentaphyllum* (Thunb.) Makino	Cucurbitaceae	Yao gai xiu	Jiao gu lan	Whole plant; root	Herb	Dahe; Shuilong	Heat-clearing and detoxifying	Orally soup	SD-472	
*Hedera nepalensis* K. Koch	Araliaceae	Ma lian man	Ni bo er chang chun teng	Stem; leaf	Shrub	Dujiang; Sanhe	Removing pathogenic heat from the blood and toxic material from the body; dispelling wind and eliminating dampness	Grinding, decoction; orally soup	SD-243	
*Hedychium flavum* Roxb.	Zingiberaceae	Jiang hua	Huang jianghua	Rhizome	Herb	Whole county	Cough	Decoction	SD-121	
*Hedyotis chrysotricha* (Palib.) Merr.	Rubiaceae	Shang kou cao	Jin mao er cao	Whole plant	Herb	Whole county	Ascaris	Grinding, decoction; orally soup	SD-406	*
*Hemiboea subcapitata* Clarke	Gesneriaceae	Ha gan dang	Ban she ju tai	Whole plant	Herb	Dahe; Shuilong	Venomous snake bite	Fresh herbs are placed on the affected area	SD-118	
*Hemsleya sphaerocarpa* Kuang et A. M. Lu	Cucurbitaceae	Bai wei lian	She lian	Root	Fungi	Dahe; Dujiang	Heat-clearing and detoxifying; eliminating dampness	Grinding, decoction; orally soup	SD-018	*
Heptapleurum delavayi *Franch*.	Araliaceae	Mei ding an	Sui xu e zhang chai	Rhizome; leaf	Tree	Jiuqian; Zhouqin	Bruises	Decoction	SD-317	
*Heptapleurum heptaphyllum* (L.) Y. F. Deng	Araliaceae	Mei ding ai	E zhang chai	Root; bark; leaf	Shrub	Jiuqian; Zhouqin	Promoting blood flow and tendon relaxation; relaxing tendon and activation collaterals	Grinding, decoction	SD-316	
*Hibiscus mutabilis* L.	Malvaceae	Dou ban lan	Mu fu rong	Flower; root; leaf	Shrub	Whole county	Inducing diuresis for removing edema; detumescence	Grinding, decoction; orally soup	SD-062	
*Hibiscus syriacus* L.	Malvaceae	Dou ban di	Mu jin	Stem; root; leaf; flower; fruit	Shrub	Sanhe; Zhouqin	Removing pathogenic heat from the blood and toxic material from the body	Grinding, decoction	SD-061	
*Houpoea officinalis* (Rehder & E. H. Wilson) N. H. Xia & C. Y. Wu	Magnoliaceae	Hou pi	Hou pu	Bark; flower; fruit	Tree	Dujiang; Jiuqian	Eliminating phlegm and stopping cough	Decoction	SD-131	*
*Houttuynia cordata* Thunb.	Saururaceae	Ma fan	Ji cai	Whole plant	Herb	Whole county	Invigorating the spleen and promotes digestion; appetizing digestion	Boiled with meat and drunk the soup	SD-192	
*Hovenia acerba* Lindl.	Rhamnaceae	Wan shou guo	Zhui ju	Bark; fruit	Tree	Dahe; Dujiang	Expelling wind-damp	Grinding, decoction; orally soup	SD-436	*
*Humulus scandens* (Lour.) Merr.	Cannabaceae	Ma ga pa	Lv cao	Whole plant	Herb	Whole county	Heat-clearing and detoxifying	Grinding, decoction	SD-194	
*Hydrangea macrophylla* (Thunb.) Ser.	Hydrangeaceae	Kun ga xiu	Xiu qiu	Root; leaf; flower	Shrub	Whole county	Antimalaria	Grinding, decoction; orally soup	SD-150	
*Hylodesmum podocarpum* subsp. *oxyphyllum* (Candolle) H. Ohashi & R. R. Mill	Fabaceae	Duo xiu ga	Jian ye chang bing shan ma huang	Root	Shrub	Whole county	Preventing further attack of malaria	Grinding, decoction	SD-073	
*Hypericum japonicum* Thunb. ex Murray	Hypericaceae	Ma ka di	Di er cao	Whole plant	Herb	Whole county	Hepatitis	Grinding, decoction; orally soup	SD-233	
*Hypericum monogynum* L.	Hypericaceae	Ma wa pai xiu	Jin si tao	Whole plant	Tree	Whole county	Heat-clearing and detoxifying; expelling wind-damp	Grinding, decoction; orally soup	SD-273	
*Hypericum sampsonii* Hance	Hypericaceae	Ma suan long	Yuan bao cao	Whole plant	Herb	Whole county	Bruises	Decoction	SD-264	
*Impatiens balsamina* L.	Balsaminaceae	Ling ma xian	Feng xian hua	Whole plant	Herb	Whole county	Promoting blood circulation; blood circulation	Decoction	SD-155	
*Imperata cylindrica* (L.) Beauv.	Poaceae	Gang yao man	Bai mao	Root; leaf; flower	Herb	Whole county	Removing pathogenic heat from the blood and toxic material from the body	Decoction	SD-099	
*Isatis tinctoria* Linnaeus	Brassicaceae	Cha lan	Song lan	Root; leaf	Herb	Whole county	Heat-clearing and detoxifying	Grinding, decoction; orally soup	SD-031	*
*Isodon amethystoides (Bentham)* H. Hara	*Lamiaceae*	Ha ke	Xiang cha cai	Whole plant	Herb	Whole county	Clearing heat and dampness, promoting blood circulation and dispersing blood stasis, detoxification and swelling	Grinding, decoction; orally soup	SD-051	
*Juglans regia* L.	Juglandaceae	Gei tao	Hu tao	Seed	Tree	Whole county	Tonifying kidney	Grinding, decoction	SD-110	
*Juniperus chinensis* L.	Cupressaceae	Nv mei an	Yuan bai	Branch	Tree	Whole county	The blood circulation hematischesis	Grinding, decoction; orally soup	SD-381	
*Justicia procumbens* Linnaeus	Acanthaceae	Ma ding	Jue chuang	Whole plant	Herb	Whole county	Promoting blood flow and tendon relaxation; back injured; waist and sinews strengthen	Grinding, decoction; orally soup	SD-178	
*Kalopanax septemlobus* (Thunb.) Koidz.	Araliaceae	Mei chang jian	Ci qiu	Bark	Tree	Dujiang; Jiuqian	Eliminating phlegm and stopping cough	Grinding, decoction; orally soup	SD-309	
*Koenigia lichiangensis* (W. W. Sm.) T. M. Schust. & Reveal	Polygonaceae	Xiang guo	Li jiang liao	Rhizome	Herb	Whole county	Blood circulation	Decoction	SD-448	
*Kyllinga brevifolia* Rottb.	Cyperaceae	Gang dong ye	Duan ye shui wu gong	Whole plant; root	Herb	Whole county	Inducing diuresis for removing edema	Decoction	SD-088	*
*Lablab purpureus* (L.) Sweet	Fabaceae	Yao duo man	Bian dou	Whole plant	Fungi	Whole county	Dispelling wind and eliminating dampness	Medicated bath	SD-469	
*Leibnitzia anandria* (Linnaeus) Turczaninow	Asteraceae	Ba di	Da ding cao	Whole plant; root	Herb	Whole county	Dispelling wind and eliminating dampness; expelling wind-damp	Decoction	SD-008	
*Leonurus japonicus* Houttuyn	Lamiaceae	Ma ka bo	Yi mu cao	Whole plant; seed	Herb	Whole county	Regulate the menstrual function to stop pain	Grinding and drink with wine	SD-232	
*Lepisorus angustus* Ching.	Polypodiaceae	Jie ge ling	Xia ye wa wei	Whole plant	Herb	Dujiang; Shuilong	Heat-clearing and detoxifying	Decoction	SD-141	
*Lepisorus bicolor* Ching.	Polypodiaceae	Pian ji wei	Er se wa wei	Whole plant	Herb	Whole county	Gastroenteritis; harmonizing stomach	Grinding, decoction	SD-389	*
*Lepisorus thunbergianus* (Kaulf.) Ching.	Polypodiaceae	Gang duo ren	Wa wei	Whole plant	Herb	Whole county	Infantile convulsion	Grinding, decoction; orally soup	SD-091	
*Leptodesmia microphylla* (Thunb.) H. Ohashi & K. Ohashi	Fabaceae	Sui mi chai	Xiao ye xi ma huang	Whole; root	Herb	Sanhe; Zhouqin	Moistening lung for arresting cough; anti-asthmatic	Grinding, decoction; orally soup	SD-416	*
*Lespedeza tomentosa* (Thunb.) Sieb	Fabaceae	Ga nai han	Rong mao hu zhi zi	Root	Shrub	Puan; Shuilong	Replenishing deficiency and replenishing qi	Boiled with meat and drunk the soup	SD-082	
*Libanotis seseloides* (Fisch. et Mey.) Turcz.	Apiaceae	Fa guo xiang cai	Xiang qin	Whole plant; fruit	Herb	Whole county	Decreasing blood pressure	Boiled with meat and drunk the soup	SD-075	*
*Ligusticum sinense* 'Chuanxiong'	Apiaceae	Ma hong di	Chuan xiong	Root; leaf	Herb	Whole county	Detumescence and promoting granulation; drainage of pus and dissolving carbuncle	Grinding, decoction; orally soup	SD-221	
*Ligustrum japonicum* Thunb.	Oleaceae	Mei ga	Ri ben nv zhen	Leaf	Shrub	Whole county	Heat-clearing and detoxifying	Boil	SD-330	*
*Lilium brownii* F. E. Brown ex Miellez	Liliaceae	Qiu ba	Ye bai he	Bulbs	Herb	Dahe; Puan	Moistening lung for arresting cough	Grinding, decoction; orally soup	SD-397	
*Lindernia crustacea* (L.) F. Muell	Linderniaceae	Ma wa gao	Mu cao	Whole plant	Herb	Puan; Zhouqin	Regulate the menstrual function to stop pain; manage qi and activating blood	Decoction	SD-269	
*Liquidambar formosana* Hance	Altingiaceae	Mei fu	Feng xiang shu	Root; leaf; fruit	Tree	Whole county	Stanch flooding	Medicinal liquor	SD-328	
*Lithospermum erythrorhizon* Sieb. et Zucc.	Boraginaceae	Ma hou	Zi cao	Root	Herb	Jiuqian; Zhouqin	Invigorating blood circulation and stopping pains	Grinding, decoction	SD-223	
*Lobelia chinensis* Lour.	Campanulaceae	Mao ao fang	Ban bian lian	Whole plant	Herb	Jiuqian; Zhouqin	Venomous snake bite	Fresh herbs are placed on the affected area	SD-298	
Lobelia davidii *Franch*.	Campanulaceae	Da zhong ban bian lian	Jiang nan shan geng cai	Root	Tree	Dujiang; Jiuqian	Inducing diuresis for removing edema	Grinding, decoction	SD-043	*
Lobelia nummularia* Lam*.	Campanulaceae	Ge zhu	Tong chui yu dai cao	Whole plant	Herb	Jiuqian; Zhouqin	Heat-clearing and detoxifying; bruises	Grinding, decoction; orally soup	SD-108	
*Lonicera japonica* Thunb.	Caprifoliaceae	Yao hua nian	Ren dong	Flower; vine	Shrub	Whole county	Heat-clearing and detoxifying	Grinding, decoction	SD-475	
*Lophatherum gracile* Brongn.	Poaceae	Tu wa fen	Dan zhu ye	Whole plant	Herb	Whole county	Removing pathogenic heat from the blood and toxic material from the body	Grinding, decoction	SD-429	
*Loropetalum chinense* Oliv*.* var. *rubrum* Yieh	Hamamelidaceae	Lu mei fei	Ji mu	Flower; rhizome	Shrub	Dujiang; Zhouqin	Hemostasis	Fresh herbs are placed on the affected area	SD-158	
*Ludwigia adscendens* (L.) Hara	Onagraceae	Guo tang she	Shuilong	Whole plant	Herb	Whole county	Heat-clearing and detoxifying	Grinding, decoction; orally soup	SD-114	*
*Ludwigia prostrata* Roxb.	Onagraceae	Shui huang ma	Ding xiang liao	Whole plant	Herb	Whole county	Eliminating phlegm and stopping cough	Decoction	SD-414	*
*Lycium barbarum* L.	Solanaceae	Ma na	Ning xia gou qi	Fruit; root	Shrub	Whole county	Nourishing liver and kidney	Medicinal liquor	SD-255	*
*Lycopodium japonicum* Thunb. ex Murray	Lycopodiaceae	Yao man jie	Shi song	Whole plant	Herb	Dujiang	Inducing diuresis for removing edema	Decoction	SD-482	
*Lygodium japonicum* (Thunb.) Sw.	Lygodiaceae	Mao nu ga	Hai jin sha	Rhizome	Fungi	Whole county	Inducing diuresis for removing edema	Grinding, decoction; orally soup	SD-299	
*Lysimachia clethroides* Duby	Primulaceae	Ma hong	Ai tao	Whole plant	Herb	Dujiang; Shuilong	Moistening lung for arresting cough; eliminating phlegm and stopping cough	Grinding, decoction; orally soup	SD-218	
*Lysimachia patungensis* Hand.-Mazz.	Primulaceae	Ma hong dang	Ba dong guo lu huang	Whole plant	Herb	Dujiang; Dahe	Analgesia	Fresh herbs are placed on the affected area	SD-220	
*Lysimachia phyllocephala* Hand.-Mazz.	Primulaceae	Ma hong	Ye tou guo luo huang	Whole plant	Herb	Dujiang; Sanhe	Eliminating phlegm and stopping cough	Grinding, decoction; orally soup	SD-219	
*Lysionotus pauciflorus* Maxim.	Gesneriaceae	Fan duo ding	Diao shi ju tai	Whole plant	Shrub	Dahe; Zhouqin	Drainage of pus and dissolving carbuncle; detumescence and promoting granulation	Grinding, decoction	SD-077	
*Macleaya cordata* (Willd.) R. Br.	Papaveraceae	Mei gan xi	Bo luo hui	Whole plant	Herb	Puan; Sanhe	Heat-clearing and detoxifying; analgesia	Fresh herbs are placed on the affected area	SD-331	
*Maclura cochinchinensis* (Loureiro) Corner	Moraceae	Wei zhi	Gou ji	Root	Shrub	Whole county	Inducing diuresis for removing edema	Grinding, decoction	SD-439	*
*Maesa japonica* (Thunb.) Moritzi. ex Zoll.	Primulaceae	Tu heng shan	Du jing shan	Rhizome; leaf	Shrub	Whole county	Inducing diuresis for removing edema	Decoction	SD-426	*
*Mahonia fortunei* (Lindl.) Fedde	Berberidaceae	Fan men man	Shi da gong lao	Whole plant	Shrub	Whole county	Antidiarrheal	Decoction	SD-078	
*Malaxis monophyllos* (L.) Sw.	Orchidaceae	Xiao zhu lan	Yuan zhao lan	Whole plant	Herb	Dahe; Sanhe	Heat-clearing and detoxifying	Grinding, decoction	SD-453	*
*Mallotus philippensis* (Lam.) Muell. Arg.	Euphorbiaceae	Hong guo guo	Cu kang chai	Root	Tree	Dahe; Dujiang	Killing taenia solium	Grinding, decoction	SD-129	*
*Malva verticillata* L.	Malvaceae	Dong kui ye kui miao	Ye kui	Whole plant;seed	Herb	Whole county	Swelling and pain in throat	Grinding, decoction	SD-057	*
*Marsilea quadrifolia* L.	Marsileaceae	Bing gong na	Ping	Whole plant	Herb	Whole county	Venomous snake bite	Fresh herbs are placed on the affected area	SD-027	
*Medicago lupulina* L.	Fabaceae	Hei jia mu xiu	Tian lan mu xu	Whole plant	Herb	Whole county	Heat-clearing and detoxifying; eliminating dampness	Grinding, decoction	SD-126	*
*Melastoma candidum* D. Don	Melastomataceae	Xia ye ye mu dan	Ye mu dan	Whole plant	Shrub	Whole county	The blood circulation hematischesis	Decoction	SD-445	*
*Melastoma dodecandrum* Lour.	Melastomataceae	Ma geng	Di ren	Whole plant	Shrub	Whole county	Expelling wind-damp	Decoction	SD-202	
*Melia azedarach* L.	Meliaceae	Mei hong	Lian	Bark; leaf;seed	Tree	Whole county	Killing parasites to relieve itching	Decoction	SD-341	
*Menyanthes trifoliata* L.	Menyanthaceae	Chuo cai	Shui cai	Whole plant	Herb	Dujiang; Jiuqian	Invigorating the spleen and promotes digestion; harmonizing stomach	Decoction	SD-038	*
*Microlepia marginata* (Houtt.) C. Chr.	Dennstaedtiaceae	Bian yuan lin jue	Bian yuan lin gai jue	Whole plant	Herb	Dujiang	Heat-clearing and detoxifying; inducing diuresis for removing edema	Decoction	SD-026	*
Microsorum insigne *(Blume) Copel.*	Polypodiaceae	Gang hai	Yu lie xing jue	Whole plant	Herb	Whole county	Inducing diuresis for removing edema	Orally soup	SD-093	
*Mimosa pudica* L.	Fabaceae	Ma xie liu	Han xiu cao	Whole plant	Herb	Whole county	Tranquilization	Grinding, decoction; orally soup	SD-281	
*Mirabilis jalapa* L.	Nyctaginaceae	Ji liu ga	Zi mo li	Tuber	Herb	Dujiang; Sanhe	Clearing liver to add yin	Grinding, decoction	SD-138	
*Miscanthus sinensis* Anderss.	Poaceae	Ba mao	Mang	Stem	Herb	Whole county	Blood circulation	Grinding, decoction; orally soup	SD-012	*
*Morella rubra* Lour.	Myricaceae	Ma kang	Yang mei	Root	Tree	Whole county	Hemostasis	Medicinal liquor	SD-236	
*Morus alba* L.	Moraceae	Mei gao dian	Sang	Leaf; root	Tree	Whole county	Eliminate the pulmonary heat	Grinding, decoction	SD-333	
*Munronia pinnata* (Wallich) W. Theobald	Meliaceae	Bang jiao gao	Yu zhuang di huang lian	Root	Shrub	Puan	Swelling and pain in throat; empyrosis	Grinding, decoction	SD-022	*
*Murdannia triquetra* (Wall. ex C. B. Clarke) Bruckn.	Commelinaceae	ma mo gai	Shui zhu ye	Whole plant	Herb	Whole county	Tonifying kidney	Decoction	SD-253	
*Mussaenda pubescens* W. T. Aiton	Rubiaceae	Mei nong an	Yu ye jin hua	Root	Shrub	Whole county	Heat-clearing and detoxifying; eliminating cold stop pain	Grinding, decoction	SD-351	
*Nandina domestica* Thunb.	Berberidaceae	Mei wa yin	Nan tian zhu	Fruit; root	Shrub	Whole county	Dysentery	Decoction	SD-357	
*Nephrolepis cordifolia* (L.) C. Presl	Nephrolepidaceae	ni ge ding	Shen jue	Tuber; leaf	Herb	Whole county	Eliminating phlegm and stopping cough	Decoction	SD-378	
*Nerium oleander* L.	Apocynaceae	Ga geng lao	Jia zhu tao	Leaf; bark	Shrub	Whole county	Promoting blood circulation	Decoction	SD-081	
*Nymphaea tetragona* Georgi	Nymphaeaceae	Lan duo	Shui lian	Flower; root	Herb	Whole county	Infantile convulsion	Grinding, decoction	SD-153	
*Odontosoria chinensis* J. Sm	Lindsaeaceae	Ma hen gong di	Wu jue	Whole plant; root	Herb	Whole county	Knife wound	Fresh herbs are placed on the affected area	SD-216	
*Ophioglossum reticulatum* L.	Ophioglossaceae	Ma bai	Xin ye ping er xiao cao	Whole plant	Herb	Whole county	Bruises; inducing diuresis for removing edema	Fresh herbs are placed on the affected area	SD-164	
*Ophioglossum thermale* Kom.	Ophioglossaceae	Ma wa ma	Xia ye ping er cao	Whole plant	Herb	Whole county	Inducing diuresis for removing edema	Grinding, decoction	SD-272	
*Ophioglossum vulgatum* L.	Ophioglossaceae	Ma bi gu	Ping er xiao cao	Whole plant	Herb	Whole county	Inducing diuresis for removing edema	Grinding, decoction	SD-168	
*Ophiopogon japonicus* (L. f.) Ker-Gawl.	Asparagaceae	Xiang yu	Mai dong	Tuber	Herb	Dahe; Sanhe; Shuilong	Eliminating phlegm and stopping cough	Grinding, decoction	SD-451	
*Origanum vulgare* L.	Lamiaceae	Ma du kong	Niu zhi	Whole plant	Shrub	Whole county	Preventing or arresting vomiting	Decoction	SD-186	
*Orixa japonica* Thunb.	Rutaceae	Mei hu	Chou chang shan	Rhizome; leaf; flower	Shrub	Dujiang; Dahe	Hepatitis; clearing liver to add yin	Medicinal liquor	SD-342	
*Orobanche coerulescens* Steph.	Orobanchaceae	Mu tong ma dou ling	Lie dang	Whole plant	Herb	Dujiang; Jiuqian	Nourishing liver and kidney	Grinding, decoction; orally soup	SD-368	*
*Osbeckia chinensis* L. ex Walp.	Melastomataceae	Bei zi cao	Jin jin xiang	Whole plant	Herb	Puan; Zhouqin	Dysentery	Grinding, decoction; orally soup	SD-024	*
*Osbeckia stellata* Ham. ex D. Don: C. B. Clarke	Melastomataceae	Gao jiao hong gang	Chao tian guan	Whole plant	Shrub	Whole county	The blood circulation hematischesis	Grinding, decoction; orally soup	SD-105	*
*Osmunda japonica* Thunb.	Osmundaceae	Gao jiao guan zhong	Zi qi	Root	Herb	Whole county	Dispelling wind and eliminating dampness	Decoction	SD-104	*
*Oxalis corniculata* L.	Oxalidaceae	Ma ao mo	Zuo jiang cao	Whole plant; root	Herb	Whole county	Removing stasis	Grinding, decoction	SD-161	
*Paederia foetida* L.	Rubiaceae	Yao de ma	Ji shi teng	Whole plant	Fungi	Whole county	Nourish yin and strengthen yang	Decoction	SD-467	
*Paeonia lactiflora* Pall.	Paeoniaceae	Ma yang	Shao yao	Root	Herb	Puan; Sanhe	Decreasing swelling to relieving pain	Grinding, decoction	SD-286	
*Palhinhaea cernua* (L.) Vasc. et Franco	Lycopodiaceae	Fa dong	Chui sui shi song	Whole plant	Herb	Dujiang	Dispelling wind and eliminating dampness	Grinding, decoction; orally soup	SD-074	
*Panax pseudoginseng Wall*	Araliaceae	Ma gou fen	Jia ren shen	Root	Herb	Dujiang; Dahe	Invigorates the spleen and promotes digestion	Grinding, decoction	SD-205	
*Paris polyphylla* Smith	Melanthiaceae	Di hui tong	Qi ye yi zhi hua	Rhizome	Herb	Dujiang; Jiuqian	Nourishing liver and kidney	Decoction	SD-048	
*Passiflora cupiformis* Mast.	Passifloraceae	Dao	Bei ye xi fan lian	Whole plant; root	Fungi	Dujiang; Jiuqian; Puan	Knife wound	Fresh herbs are placed on the affected area	SD-046	
*Patrinia villosa* (Thunb.) Juss.	Caprifoliaceae	Ma gan ga	Pan dao zeng	Whole plant	Herb	Whole county	Heat-clearing and detoxifying	Grinding, decoction	SD-195	
*Pedicularis labordei* Vant. ex Bonati	Orobanchaceae	La shi ma xian hao	Xi nan ma xian hao	Whole plant	Herb	Whole county	Heat-clearing and detoxifying	Grinding, decoction; orally soup	SD-152	*
*Perilla frutescens* (L.) Britt.	Lamiaceae	An ga	Zi su	Leaf; seed	Tree	Dahe; Sanhe; Puan	Regulate qi; tocolysis	Grinding, decoction	SD-003	
*Periploca calophylla* (Wight) Falc.	Apocynaceae	Hei gu tou	Qing she teng	Stem	Shrub	Dujiang; Jiuqian	Bruises; strong bones and muscles	Decoction	SD-125	*
*Peristrophe japonica* (Thunb.) Bremek.	Acanthaceae	Ma wa lan	Jiu tou shi zi cao	Whole plant	Herb	Whole county	Appetizing digestion; dispelling wind	Decoction	SD-270	
*Persicaria chinensis* (L.) H. Gross	Polygonaceae	Ao meng ga man	Huo tan mu	Whole plant	Herb	Whole county	Heat-clearing and detoxifying	Grinding, decoction	SD-006	
*Persicaria filiformis* (Thunb.) Nakai	Polygonaceae	Chong yang liu	Jin xian cao	Whole plant	Herb	Dahe; Dujiang	Inflammation	Grinding, decoction; orally soup	SD-035	*
*Persicaria hydropiper* (L.) Spach	Polygonaceae	Ma fan	Shui liao	Whole plant	Herb	Whole county	Inducing diuresis for removing edema	Decoction	SD-193	
*Persicaria perfoliata* (L.) H. Gross	Polygonaceae	Ma o ding	Kang ban gui	Stem; leaf	Herb	Whole county	Moistening lung for arresting cough	Decoction	SD-260	
*Persicaria tinctoria* (Aiton) Spach	Polygonaceae	Hong la liao	Liao lan	Leaf	Herb	Whole county	Heat-clearing and detoxifying	Decoction	SD-130	*
*Peucedanum praeruptorum* Dunn	Apiaceae	Ma hai	Qian hu	Root	Herb	Dujiang; Jiuqian	Eliminating phlegm and stopping cough	Grinding, decoction	SD-214	
*Phegopteris decursive-pinnata* (H. C. Hall) Fée	Thelypteridaceae	Xia yu jin xing jue	Yan yu luan guo jue	Leaf	Herb	Dujiang	Inducing diuresis for removing edema; eliminating dampness	Decoction	SD-446	*
*Phellodendron chinense* Schneid	Rutaceae	Gei mei xiang	Chuan Huang po	Bark	Tree	Dahe; Jiuqian	Clearing liver to add yin	Grinding, decoction	SD-109	
*Phoebe sheareri* (Hemsl.) Gamble	Lauraceae	Zi jin nan	Zi nan	Root; leaf	Tree	Dujiang; Jiuqian	Harmonizing stomach	Grinding, decoction	SD-503	*
*Pholidota chinensis* Lindl.	Orchidaceae	Pang bao dui	Shi xian tao	Tuber	Tree	Whole county	Asthma	Decoction	SD-386	
*Phragmites australis* (Cav.) Trin. ex Steud.	Poaceae	Hao lu su	Lu wei	Root	Herb	Whole county	Moistening lung for arresting cough; deficiency of body fluids	Grinding, decoction	SD-123	*
*Phyla nodiflora* (L.) Greene	Caprifoliaceae	Shuilong	Guo jiang teng	Whole plant	Herb	Whole county	Dysentery; preventing further attack of malaria	Grinding, decoction	SD-415	*
*Phyllanthus urinaria* L.	Phyllanthaceae	Duo guo ga	Ye xia zhu	Whole plant	Herb	Dujiang; Dahe	Improving eyesight and removing nebula; heat-clearing and detoxifying	Decoction	SD-070	
*Phytolacca acinosa* Roxb.	Phytolaccaceae	Mei ma lang	Shang lu	Root; flower	Herb	Whole county	Heat-clearing and detoxifying; bruises	Grinding, decoction	SD-349	
*Pilea notata* C. H. Wright	Urticaceae	Ma wa zu	Leng shui hua	Whole plant	Herb	Whole county	Heat-clearing and detoxifying; clearing heat; dehumidification	Decoction	SD-274	
*Pinellia pedatisecta* Schott	Araceae	Nan bo da	Hu zhang	Tuber	Herb	Dujiang; Sanhe	Eliminating phlegm and stopping cough	Grinding, decoction	SD-372	
*Pinellia ternata* (Thunb.) Breit.	Araceae	Di hui xi	Ban xia	Tuber	Herb	Whole county	Eliminating phlegm and stopping cough	Grinding, decoction	SD-049	
*Pinus massoniana* Lamb.	Pinaceae	Bu mai suo	Ma wei song	Leaf	Tree	Whole county	Dispelling wind and eliminating dampness	Decoction	SD-029	
*Piper wallichii* (Miq.) Hand.-Mazz.	Piperaceae	Ma mei	Shi nan teng	Whole plant	Fungi	Whole county	Eliminating phlegm and cough suppressant	Grinding, decoction	SD-247	
*Pittosporum tobira* (Thunb.) Ait.	Pittosporaceae	Dong yu ga	Hai tong	Root; seed; leaf	Shrub	Dahe; Sanhe	Relieving dryness and moistening; tranquilization	Grinding, decoction	SD-059	
*Plantago asiatica* L	Plantaginaceae	Ma ma pa	Che qian	Whole plant	Herb	Whole county	Eliminating phlegm and stopping cough	Grinding, decoction	SD-246	
*Platycladus orientalis* (L.) Franco	Cupressaceae	Nv mei ou	Ce bai	Branch; seed	Tree	Whole county	Gastroenteritis	Decoction	SD-383	
*Platycodon grandiflorus* (Jacq.) A. DC.	Campanulaceae	Xiang dian	Jie geng	Root	Herb	Whole county	Eliminating phlegm and stopping cough	Decoction	SD-447	
*Pleione bulbocodioides* (Franch.) Rolfe	Orchidaceae	Tiao zi qi	Du suan lan	Stem	Herb	Dahe; Dujiang	Eliminating phlegm and stopping cough	Decoction	SD-422	*
*Pleuropterus multiflorus* (Thunb.) Nakai	Polygonaceae	Man gang xi	He shou wu	Tuber; vine	Fungi	Whole county	Nourishing liver and kidney; regulate qi	Orally soup	SD-295	
*Polycarpaea corymbosa* (L.) Lamarck	Caryophyllaceae	Ma san dang	Bai gu ding	Whole plant	Herb	Sanhe; Zhouqin	Eczema; relieving rheumatism and cold	Grinding, decoction; orally soup	SD-263	
*Polygala japonica* Houtt.	Polygalaceae	Dong yao dong	Gua zi jin	Whole plant; root	Herb	Whole county	Eliminating phlegm and stopping cough	Grinding, decoction	SD-058	
*Polygala sibirica* L.	Polygalaceae	Ma dian di	Xi bo li ya yuan zhi	Whole plant	Herb	Whole county	Eliminating phlegm and stopping cough; tranquilization	Grinding, decoction	SD-177	
*Polygonatum cirrhifolium* (Wall.) Royle	Asparagaceae	Ma xin meng	Juan ye huang jing	Rhizome	Herb	Dujiang; Jiuqian	Nourishing yin and tonifying yang	Grinding, decoction	SD-282	
*Polygonatum odoratum* (Mill.) Druce	Asparagaceae	Ma ding man	Yu zhu	Rhizome	Herb	Dujiang; Sanhe	Moistening lung for arresting cough	Decoction	SD-183	
*Polygonum aviculare* L.	Polygonaceae	Pian xu	Pian xu	Whole plant	Herb	Whole county	Inflammation	Grinding, decoction; orally soup	SD-390	*
*Populus davidiana* Dode	Salicaceae	Da ye yang	Shan yang	Bark	Tree	Whole county	Ascaris	Grinding, decoction; orally soup	SD-042	*
*Poria cocos* (Schw.) Wolf.	Polyporaceae	Ni ga	Fu ling	Sclerotia	Herb	Whole county	Strengthen the spleen; tranquilization; inducing diuresis for removing edema	Decoction	SD-376	
*Portulaca oleracea* L.	Portulacaceae	Ma wa fa	Ma chi xian	Whole plant	Herb	Dujiang; Sanhe	Dysentery	Grinding, decoction	SD-267	
*Potentilla chinensis* Ser.	Rosaceae	Ka gui di	Wei ling cai	Whole plant; root	Herb	Whole county	Heat-clearing and detoxifying	Grinding, decoction; orally soup	SD-147	
*Potentilla freyniana* Bornm.	Rosaceae	Ma ding man	San ye wei ling cai	Whole plant; root	Herb	Whole county	Regulating the menstrual function to stop pain; checking vaginal discharge	Decoction	SD-184	
*Prunella vulgaris* L.	Lamiaceae	Ma ding ma	Xia ku cao	Whole plant; fruit	Herb	Whole county	Clearing liver to add yin; clearing heat for detumescence	Decoction	SD-182	
*Prunus armeniaca* L.	Rosaceae	Mv mei feng	Xing	Seed	Tree	Dahe; Puan	Moistening lung for arresting cough	Grinding, decoction; orally soup	SD-370	
*Prunus mume* Siebold & Zucc.	Rosaceae	Qing mei	Mei	Root; flower; fruit	Tree	Jiuqian; Puan	Dysentery	Orally soup	SD-395	
*Prunus persica* L.	Rosaceae	Nv fang	Tao	Seed	Tree	Whole county	Promoting blood flow and tendon relaxation	Decoction	SD-380	
Prunus pseudocerasus* (Lindl.) G. Don*	Rosaceae	Du fang	Ying tao	Fruit; root	Tree	Whole county	Drainage of pus and dissolving carbuncle	Medicinal liquor	SD-067	
*Pseudognaphalium affine* (D. Don) Anderberg	Asteraceae	Ma gan geng	Shu qu cao	Whole plant	Herb	Whole county	Eliminating phlegm and stopping cough; asthma	Decoction	SD-196	
*Pteridium aquilinum* var. *latiusculum* (Desv.)Underw.ex Heller	Dennstaedtiaceae	Quan tou cai	Jue	Whole plant	Herb	Whole county	Expelling wind-damp; heat-clearing and detoxifying	Decoction	SD-399	*
*Pteris cretica* L. var. *nervossa* (Thunb.) Ching et S.H. Wu	Pteridaceae	You hen gong	Feng wei jue	Whole plant	Herb	Whole county	Removing pathogenic heat from the blood and toxic material from the body	Grinding, decoction; orally soup	SD-496	
*Pteris dispar* Kze.	Pteridaceae	Ban bian qi	Ci chi ban bian qi	Whole plant	Herb	Whole county	Laxative	Grinding, decoction; orally soup	SD-020	*
*Pteris vittata* L. f	Pteridaceae	Ma you du ku	Wu gong feng wei jue	Whole plant	Herb	Whole county	Disinfection	Medicinal liquor	SD-288	
*Pterocarya stenoptera* C. DC.	Juglandaceae	Mei lou wen	Feng yang	Bark; leaf	Tree	Dujiang; Sanhe	Disinfection; killing parasites to relieve itching	Grinding, decoction; orally soup	SD-348	
*Pueraria edulis* Pampan.	Fabaceae	Ge	Shi yong ge	Root; flower	Fungi	Whole county	Dysentery	Grinding, decoction; orally soup	SD-106	*
*Pueraria montana* var. *lobata* (Willdenow) Maesen & S. M. Almeida ex Sanjappa & Predeep	Fabaceae	Yao hai	Ge	Tuber	Fungi	Whole county	Eczema; dispelling wind and eliminating dampness	Grinding, decoction; orally soup	SD-474	
*Puhuaea sequax* (Wall.) H. Ohashi & K. Ohashi	Fabaceae	Ma wa diu	Wa zi cao	Whole plant; root	Shrub	Whole county	Antivirus and insect repellent	Grinding, decoction	SD-266	
*Punica granatum* L.	Lythraceae	Suo liu	Shi liu	Fruit; root; leaf; flower	Tree	Whole county	Dysentery	Decoction	SD-417	
*Pyrola calliantha* H. Andr.	Ericaceae	Ma yan duo	Lu ti cao	Whole plant	Shrub	Dujiang; Zhouqin	Moistening lung for arresting cough	Grinding, decoction; orally soup	SD-285	
*Pyrrosia lingua* (Thunb.) Farwell	Polypodiaceae	Ma mo hui	Shi wei	Whole plant	Herb	Dujiang; Shuilong	Heat-clearing and detoxifying; clearing heat; dehumidification	Decoction	SD-254	
*Pyrrosia piloselloides (Linnaeus)* M. G. Price	Polypodiaceae	Ma gao hui	Bao shu lian	Whole plant	Herb	Dujiang; Lalan	Removing pathogenic heat from the blood and toxic material from the body	Orally soup	SD-197	
*Pyrrosia similis* Ching	Polypodiaceae	Ha jie ge	Xiang si shi wei	Whole plant; root	Herb	Dujiang; Shuilong	Inducing diuresis for removing edema	Decoction	SD-119	
*Pyrus ussuriensis* Maxim	Rosaceae	Gei xiu	Qi zi li	Fruit	Tree	Whole county	Harmonizing stomach	Decoction	SD-111	
*Quercus acutissima* Carr.	Fagaceae	Gao ding	Ma li	Fruit	Tree	Whole county	Gastroenteritis	Grinding, decoction; orally soup	SD-103	
*Quercus fabri* Hance	Fagaceae	Za wa lao	Bai li	Fruit	Tree	Whole county	Gastroenteritis; harmonizing stomach	Grinding, decoction	SD-500	
*Ranunculus japonicus* Thunb.	Ranunculaceae	Ma ding meng	Mao gen	Whole plant; root	Herb	Whole county	Gastroenteritis	Grinding, decoction	SD-185	
*Raphanus sativus* L.	Brassicaceae	Ma xiang lan	Luo bo	Seed; root; leaf	Herb	Whole county	Eliminating phlegm and stopping cough	Decoction	SD-280	
*Reineckea carnea (Andrews) Kunth*	Asparagaceae	Ma hui xiu	Ji xiang cao	Whole plant	Herb	Dahe; Zhouqin; Dujiang	Fracture; strong bones and muscles	Grinding, decoction	SD-224	
*Rhapis excelsa* (Thunb.) Henry ex Rehd.	Arecaceae	Mei yi de	Zong zhu	Root	Shrub	Dujiang; Shuilong	Inflammation	Grinding, decoction; orally soup	SD-363	
*Rhododendron simsii* Planch.	Ericaceae	Nu yao han	Du jian	Whole plant	Shrub	Dujiang; Sanhe	The blood circulation hematischesis	Decoction	SD-379	
*Rhus chinensis* Mill.	Anacardiaceae	Mei bu geng	Yan fu mu	Root	Tree	Whole county	Inflammation	Grinding, decoction; orally soup	SD-308	
*Robinia pseudoacacia* L.	Fabaceae	An lai di	Ci huai	Flower	Tree	Whole county	Inducing diuresis for removing edema; the blood circulation hematischesis	Decoction	SD-004	
*Rohdea japonica* (Thunb.) Roth	Asparagaceae	Gang ao mie	Wan nian qing	Whole plant; root	Herb	Dujiang; Jiuqian	Harmonizing stomach; appetizing digestion	Decoction	SD-084	
*Rorippa indica* (L.) Hiern	Brassicaceae	Ma nian	Han cai	Whole plant	Herb	Sanhe; Shuilong	Eliminating phlegm and stopping cough	Grinding, decoction; orally soup	SD-258	
*Rosa laevigata* Michx.	Rosaceae	Dou pang ya	Jin ying zi	Fruit; root	Shrub	Whole county	Tonifying kidney	Decoction	SD-064	
*Rosa roxburghii* Tratt.	Rosaceae	Pang ka	Sao si hua	Fruit; root	Shrub	Jiuqian; Shuilong; Zhouqin	Harmonizing stomach	Grinding, decoction; orally soup	SD-387	
*Rubia cordifolia* L.	Rubiaceae	Yao yi	Qian cao	Root	Fungi	Whole county	Removing pathogenic heat from the blood and toxic material from the body	Decoction	SD-488	
*Rubus corchorifolius* L. f	Rosaceae	Shu mei	Shan mei	Root	Shrub	Whole county	Blood circulation	Decoction	SD-413	*
*Rubus coreanus* Miq.	Rosaceae	Dou ba	Cha tian biao	Root	Shrub	Whole county	Tonifying kidney	Decoction	SD-060	
*Rubus lambertianus* Ser.	Rosaceae	Shi yue miao	Gao liang biao	Root	Shrub	Whole county	Removing stasis	Grinding, decoction; orally soup	SD-412	*
*Rubus pluribracteatus* L. T. Lu & Boufford	Rosaceae	Dong ga	Da wu biao	Root	Shrub	Whole county	Eliminating phlegm and stopping cough	Grinding, decoction; orally soup	SD-056	
*Rubus quinquefoliolatus* Yü et Lu	Rosaceae	Yao zhenng wo wa	Wu ye xuan gou zi	Whole plant	Shrub	Whole county	Bruises; heat-clearing and detoxifying	Decoction	SD-489	
*Rumex acetosa* L.	Polygonaceae	Ma hong hai	Suan mo	Root	Herb	Dujiang; Dahe	Have a laxative effect	Decoction	SD-222	
*Rumex japonicus* Houtt.	Polygonaceae	Ma zai di	Yang ti	Whole plant	Herb	Whole county	Have a laxative effect; relieving dryness and moistening	Grinding, decoction; orally soup	SD-291	
*Rumex nepalensis* Spreng	Polygonaceae	Ma da miao	Ni bo er suan mo	Whole plant	Herb	Dujiang; Dahe	Heat-clearing and detoxifying	Decoction	SD-171	
*Sagina japonica* (Sw.) Ohwi	Caryophyllaceae	Gang neng fa	Qi gu cao	Whole plant	Herb	Dujiang; Dahe	Eliminating phlegm and stopping cough	Grinding, decoction	SD-097	
*Salix babylonica* L.	Salicaceae	Mei liu	Chui liu	Branch	Tree	Whole county	Dispelling wind and eliminating dampness; dehumidification	Grinding, decoction	SD-346	
*Salix wallichiana* Anderss.	Salicaceae	Mei bai la	Zao liu	Root; leaf	Shrub	Whole county	Dispelling wind and eliminating dampness	Grinding, decoction; orally soup	SD-303	
*Salvia cavaleriei* Lévl.	Lamiaceae	Fan bei hong	Gui zhou shu wei cao	Whole plant	Herb	Whole county	The blood circulation hematischesis	Grinding, decoction; orally soup	SD-076	*
*Sambucus williamsii* Hance	Adoxaceae	Yao hui xing	Jie gu mu	Root; leaf	Shrub	Whole county	Bruises	Grinding, decoction	SD-476	
*Sanguisorba officinalis* L.	Rosaceae	Huang gua xiang	Di yu	Root	Herb	Whole county	Bleeding stop	Decoction	SD-134	*
*Sanicula orthacantha* S. Moore	Apiaceae	Ma ding ang	Zhi ci bian dou cai	Whole plant	Herb	Whole county	Harmonizing stomach; dysentery	Grinding, decoction; orally soup	SD-179	
*Sapindus saponaria* Linnaeus	Sapindaceae	Mu wan zi	Wu huan zi	Root; fruit	Tree	Dahe	Eliminating phlegm and stopping cough	Grinding, decoction; orally soup	SD-369	*
*Sarcandra glabra* (Thunb.) Nakai	Chloranthaceae	Mei han lan	Cao shan hu	Branch	Shrub	Whole county	Fracture	Grinding, decoction	SD-340	
*Sargentodoxa cuneata* (Oliv.) Rehd. et Wils.	Lardizabalaceae	Yao e nong	Da xue teng	Rhizome	Fungi	Dujiang; Jiuqian	Strong bones and muscles	Grinding, decoction; orally soup	SD-470	
*Sassafras tzumu* (Hemsl.) Hemsl.	Lauraceae	Ca shu	Cha mu	Root	Tree	Dujiang; Jiuqian	Dispelling wind and eliminating dampness	Decoction	SD-030	*
*Sauromatum giganteum* (Engler) Cusimano & Hetterscheid	Araceae	Di shui shen	Du jiao lian	Tuber	Herb	Dujiang; Sanhe	Facial paralysis	Grinding, decoction	SD-052	*
*Saururus chinensis* (Lour.) Baill.	Saururaceae	Ne jiu nan	San bai cao	Whole plant	Herb	Whole county	Detumescence; heat-clearing and detoxifying; inducing diuresis for removing edema	Grinding, decoction	SD-374	
*Saxifraga stolonifera* Curt.	Saxifragaceae	Ma da yong	Hu er cao	Whole plant	Herb	Whole county	Removing pathogenic heat from the blood and toxic material from the body	Grinding, decoction	SD-174	
*Sceptridium ternatum* (Thunb.) Y. X. Lin	Ophioglossaceae	Wa you gu	Yin di jue	Whole plant	Herb	Dujiang; Dahe	Hepatitis; infantile convulsion	Orally soup; pound fresh part applied on the affected area	SD-435	
*Schisandra chinensis* (Turcz.) Baill.	Schisandraceae	Bei wu wei zi	Wu wei zi	Fruit; root	Fungi	Dahe; Dujiang	Moistening lung for suppressing cough	Decoction	SD-023	*
*Schizophragma integrifolium* Oliv.	Hydrangeaceae	Tong ye teng	Zuan di feng	Root; vine	Fungi	Whole county	Expelling wind and activating blood flow	Orally soup	SD-425	*
*Schoenoplectiella wallichii* (Nees) Lye	Cyperaceae	Shan ji wei cao	Zhu mao cao	Whole plant	Herb	Whole county	Inducing diuresis for removing edema	Grinding, decoction; orally soup	SD-404	*
*Scleromitrion diffusum* (Willd.) R. J. Wang	Rubiaceae	Gang mo hui	Bai hua she she cao	Whole plant	Herb	Whole county	Heat-clearing and detoxifying	Grinding, decoction	SD-096	
*Scutellaria barbata* D. Don	Lamiaceae	Yang long	Ban zhi lian	Whole plant	Herb	Jiuqian; Zhouqin	Anti-inflammatory	Grinding, decoction	SD-464	
*Scutellaria indica* L.	Lamiaceae	Ma mei feng	Han xin cao	Whole plant	Herb	Dujiang; Puan	Moistening lung for arresting cough	Decoction	SD-249	
*Sedum sarmentosum* Bunge	Crassulaceae	Ma nv bu	Chui pen cao	Whole plant	Herb	Whole county	The blood circulation hematischesis	Grinding, decoction; orally soup	SD-259	
*Selaginella delicatula* (Desv.) Alston	Selaginellaceae	Shan bai zhi	Bao ye juan bai	Whole plant	Herb	Dujiang	Inducing diuresis for removing edema	Grinding, decoction	SD-402	*
*Selaginella labordei* Hieron. ex Christ	Selaginellaceae	Shan bai zhi	Xi ye juan bai	Whole plant	Herb	Jiuqian	Inducing diuresis for removing edema	Grinding, decoction	SD-403	*
*Selaginella moellendorffii* Hieron.	Selaginellaceae	Shi bai	Jiang nan juan bai	Whole plant	Herb	Dujiang	Inducing diuresis for removing edema; heat-clearing and detoxifying	Grinding, decoction; orally soup	SD-410	*
*Selaginella tamariscina* (P. Beauv.) Spring	Selaginellaceae	Ding meng bian	Juan bai	Whole plant	Herb	Dujiang; Sanhe	Hemostasis; the blood circulation hematischesis	Decoction	SD-055	
*Selaginella uncinata* (Desv.) Spring	Selaginellaceae	Gang heng men	Cui yun cao	Whole plant	Herb	Dujiang; Jiuqian	Dispelling wind and eliminating dampness	Grinding, decoction; orally soup	SD-094	
*Selliguea hastata* (Thunberg) Fraser-Jenkins	Polypodiaceae	Gang ding an	Jin ji jiao jia liu jue	Whole plant	Herb	Dujiang	Eliminating phlegm and stopping cough	Grinding, decoction; orally soup	SD-086	
*Semiaquilegia adoxoides* (DC.) Makino	Ranunculaceae	Ma gei nuo	Tian kui	Tuber	Herb	Whole county	Relieving asthma	Grinding, decoction; orally soup	SD-201	
*Senecio scandens* Buch.-Ham. ex D. Don	Asteraceae	Ma suo li	Qian li guang	Whole plant	Herb	Jiuqian; Sanhe	Expelling wind-damp	Decoction	SD-265	
*Senna tora* (L.) Roxb.	Fabaceae	Ge li nuo	Jue ming	Whole plant	Herb	Whole county	Regulate qi; promoting flow of qi and blood circulation	Grinding, decoction; orally soup	SD-107	
*Senna tora* (Linnaeus) Roxburgh	Fabaceae	Duo hen duan	Jue ming	Seed	Shrub	Whole county	Hepatitis; have a laxative effect	Grinding, decoction; orally soup	SD-072	
*Serissa serissoides* (DC.) Druce	Rubiaceae	Ma leng ga	Bai ma gu	Whole plant; root	Shrub	Sanhe; Shuilong	Clearing liver to add yin	Grinding, decoction; orally soup	SD-242	
*Sida rhombifolia* L.	Malvaceae	Huang hua di tao hua	Bai bei huang hua nian	Root; leaf	Shrub	Dahe; Sanhe	Detumescence and promoting granulation	Fresh herbs are placed on the affected area	SD-135	*
*Sigesbeckia pubescens* (Makino) Makino	Asteraceae	Du ge ma	Xian geng xi xian	Whole plant	Herb	Whole county	Nourishing liver and kidney	Orally soup	SD-068	
*Sinosenecio oldhamianus* (Maxim.) B. Nord.	Asteraceae	Ma xuan dong	Pu er gen	Whole plant	Herb	Dujiang; Sanhe	Heat-clearing and detoxifying	Grinding, decoction; orally soup	SD-283	
*Siphonostegia chinensis* Benth.	Orobanchaceae	Ma jie lu	Yin xing cao	Whole plant	Herb	Sanhe; Zhouqin	Expelling wind-damp	Grinding, decoction; orally soup	SD-228	
*Smilax glabra* Roxb.	Smilacaceae	Ni ga	Tu fu ling	Root	Shrub	Whole county	Invigorating the spleen and promotes digestion; harmonizing stomach	Grinding, decoction	SD-377	
*Solanum violaceum* Ortega	Solanaceae	Ma du meng	Ci tian qie	Fruit; leaf	Shrub	Whole county	Inducing diuresis for removing edema; detumescence	Grinding, decoction; orally soup	SD-189	
*Solena heterophylla* Lour.	Cucurbitaceae	Jie du cao	Mao gua	Root	Herb	Dahe; Dujiang	Heat-clearing and detoxifying	Boiled with meat and drunk the soup	SD-140	*
*Solidago decurrens* Lour.	Asteraceae	Ma bo man	Yi zhi huang hua	Whole plant	Herb	Whole county	Knife wound	Fresh herbs are placed on the affected area	SD-169	
*Sophora flavescens* Alt.	Fabaceae	Mei duo hong	Ku shen	Root; seed	Tree	Dahe; Sanhe	Dispelling wind and eliminating dampness	Grinding, decoction	SD-323	
*Spatholobus suberectus* Dunn	Fabaceae	Yao lang	Mi hua dou	Seed	Fungi	Dujiang; Sanhe	Clearing and activating the channels and collaterals	Orally soup	SD-479	
*Sphagnum palustre* L.	Sphagnaceae	Mian hua cai	Ni tan xian	Whole plant	Herb	Dahe; Sanhe	Heat-clearing and detoxifying	Grinding, decoction; medicated bath	SD-366	*
*Spiraea japonica* L. f.	Rosaceae	Kun ga	Fen hua xiu xian ju	Root	Shrub	Dujiang; Zhouqin	Eliminating phlegm and stopping cough	Grinding, decoction	SD-149	
*Spiranthes sinensis* (Pers.) Ames	Orchidaceae	Zhu bian cao	Shou cao	Whole plant	Herb	Whole county	Nourishing liver and kidney; tranquilization	Decoction	SD-502	*
*Stachys sieboldii* Miq.	Lamiaceae	Di li	Gan lu zi	Whole plant	Herb	Puan; Sanhe	Heat-clearing and detoxifying	Decoction	SD-050	*
*Stellaria aquatica* (L.) Scop.	Caryophyllaceae	Ma ge ling	E chang cai	Whole plant; root	Herb	Whole county	Expelling wind-damp	Fresh herbs are placed on the affected area	SD-199	
*Stellaria media* (L.) Villars	Caryophyllaceae	Ma ni	Fan lv	Stem; leaf	Herb	Whole county	Killing parasites to relieve itching; antibiosis	Grinding, decoction; orally soup	SD-256	
*Stephania cepharantha* Hay.	Menispermaceae	Ha bo	Jin xian diao wu gui	Tuber	Shrub	Dujiang; Jiuqian	Venomous snake bite	Fresh herbs are placed on the affected area	SD-115	
*Strobilanthes cusia* (Nees) Kuntze	Acanthaceae	Ban lan gen	Ban lan	Whole plant	Herb	Whole county	Clearing heat and detoxifying, reducing swelling and relieving pain	Decoction	SD-021	*
*Styrax japonicas* Sieb. & Zucc.	Styracaceae	Mei lang gui	Ye mo li	Leaf; fruit	Shrub	Dujiang	Expelling wind-damp	Grinding, decoction	SD-344	
*Symplocos lancifolia* Sieb. et Zucc.	Symplocaceae	Pi zhen ye shan fan	Guang ye shan fan	Root; leaf	Tree	Dujiang	Heat-clearing and detoxifying; bruises	Grinding, decoction	SD-388	*
*Taraxacum mongolicum* Hand.-Mazz.	Asteraceae	Ba hai	Pu gong ying	Whole plant	Herb	Whole county	Heat-clearing and detoxifying	Decoction	SD-009	
*Taxus wallichiana* var. *chinensis* (Pilger) Florin	Taxaceae	Mei fa nuo	Hong dou shan	Seed	Tree	Dujiang; Jiuqian	Gastroenteritis	Stir fry; grinding, decoction	SD-325	
*Tetrapanax papyrifer* (Hook.) K. Koch	Araliaceae	Mei bu feng	Tong tuo mu	Stem	Shrub	Whole county	Regulate the menstrual function to stop pain; lactogenesis	Grinding, decoction	SD-307	
*Tetrastigma formosanum* (Hemsl.) Gagnep.	Vitaceae	Yin ya gui	Tau wan ya pa teng	Root	Fungi	Whole county	The blood circulation hematischesis	Grinding, decoction	SD-494	
*Tetrastigma hemsleyanum* Diels et Gilg	Vitaceae	Ma gou han wa	San ye ya pa teng	Whole plant; tuber	Fungi	Dujiang; Dahe	Dispelling wind and eliminating dampness	Grinding, decoction	SD-206	
*Tetrastigma serrulatum* (Roxb.) Planch.	Vitaceae	Yao pan	Xia ye yan ya teng	Whole plant	Fungi	Dujiang; Jiuqian	The blood circulation hematischesis	Decoction	SD-486	
*Thalictrum ichangense* Lecoy. ex Oliv.	Ranunculaceae	Ma deng ren	Dun ye tang song cao	Whole plant	Herb	Dahe; Zhouqin	Expelling wind-damp	Grinding, decoction	SD-175	
*Thesium chinense* Turcz.	Santalaceae	Bai ru cao	Bai rui cao	Whole plant	Herb	Whole county	Regulate qi	Decoction	SD-017	*
*Tinospora sagittata* (Oliv.) Gagnep.	Menispermaceae	Ha jiu peng	Qing niu dan	Tuber	Fungi	Dujiang; Jiuqian	Inflammation	Grinding, decoction	SD-120	
*Toddalia asiatica* (L.) Lam.	Rutaceae	Mei ao gan	Fei long zhang xue	Root; leaf	Tree	Dujiang; jiu long	Removing stasis	Medicinal liquor	SD-302	
*Toona sinensis* (A. Juss.) Roem.	Meliaceae	Mei han ga	Xiang chun	Bark	Tree	Whole county	Dispelling wind and eliminating dampness	Grinding, decoction	SD-339	
*Torilis scabra* (Thunb.) DC.	Apiaceae	He shi	Qie yi	Whole plant	Herb	Whole county	Relieving rheumatism and cold	Grinding, decoction; orally soup	SD-124	*
*Torricellia angulata* Oliv.	Torricelliaceae	Da jie gu dan	Jiao ye qiao bing mu	Root; leaf; flower	Shrub	Whole county	Fracture	Grinding, decoction	SD-041	*
*Toxicodendron succedaneum* (L.) O. Kuntze	Anacardiaceae	Mei da	Shan qi shu	Root; leaf	Shrub	Whole county	Bruises; heat-clearing and detoxifying	Grinding, decoction; orally soup	SD-310	
*Trachelospermum jasminoides* (Lindl.) Lem.	Apocynaceae	Wang ba hai	Luo shi	Stem; leaf	Fungi	Whole county	Inflammation	Grinding, decoction	SD-437	
*Trachycarpus fortunei* (Hook.) H. Wendl.	Arecaceae	Mei yi	Zong lv	Leaf	Tree	Dujiang; Shuilong	Dysentery; preventing further attack of malaria	Grinding, decoction; orally soup	SD-362	
*Trichosanthes cucumeroides* (Ser.) Maxim.	Cucurbitaceae	Bu ga	Wang gua	Fruit; root	Fungi	Whole county	The blood circulation hematischesis	Grinding, decoction; orally soup	SD-028	
*Trigastrotheca stricta* (L.) Thulin	Molluginaceae	Mei fan hen	Su mi cao	Whole plant	Herb	Whole county	Antidiarrheal; dysentery	Decoction	SD-326	
*Typha angustifolia* L.	Typhaceae	Xiang pu	Shui zhu	Flower	Herb	Whole county	The blood circulation hematischesis	Decoction	SD-450	*
*Ulmus pumila* L.	Ulmaceae	Bai yu	Yu	Bark; root	Tree	Whole county	Checking vaginal discharge; tranquilization	Grinding, decoction; orally soup	SD-019	*
*Uncaria rhynchophylla* (Miq.) Miq. ex Havil.	Rubiaceae	Mei xiang xiu	Gou teng	Aboveground part	Fungi	Whole county	Clearing liver to add yin	Grinding, decoction; orally soup	SD-358	
*Urena labata Linn.* var. *chinensis* (Osbeck) S. Y. Hu Fl	Malvaceae	Ma ka dun	Zhong hua di tao hua	Whole plant; root	Shrub	Whole county	Dispelling wind and eliminating dampness	Grinding, decoction	SD-234	
*Usnea diffracta* Vain.	Lichenes	Po jing song luo	Song luo	Whole plant	Herb	Dujiang; Jiuqian	Dispelling wind and eliminating dampness; regulate the menstrual function to stop pain	Grinding, decoction; orally soup	SD-391	*
*Vaccinium bracteatum* Thunb.	Ericaceae	Ran shu	Nan zhu	Leaf; fruit	Shrub	Whole county	Killing parasites to relieve itching	Grinding, decoction; orally soup	SD-400	*
*Valeriana jatamansi* Jones	Caprifoliaceae	Ha fa	Zhi zhu xiang	Rhizome	Herb	Jiuqian; Puan	Expelling wind-damp	Grinding, decoction; orally soup	SD-117	
*Valeriana officinalis* L.	Caprifoliaceae	Chuan xin pai cao	Xie cao	Root	Herb	Whole county	Abdominal pain	Decoction	SD-037	*
*Verbena officinalis* L.	Verbenaceae	Ma ou	Ma bian cao	Stem	Herb	Dahe; Sanhe	Blood circulation	Grinding, decoction; orally soup	SD-261	
*Vernicia fordii* (Hemsl.) Airy-Shaw	Euphorbiaceae	Mei duo	You tong	Root; leaf; seed	Tree	Whole county	Killing parasites to relieve itching	Boiled with meat and drunk the soup	SD-322	
*Veronica anagallis-aquatica* L.	Plantaginaceae	Ma ge ling	Bei shui ku mai	Whole plant	Herb	Whole county	Regulate the menstrual function to stop pain	Decoction	SD-200	
*Veronica peregrina* L.	Plantaginaceae	Ma du nei	Fen mu cao	Whole plant	Herb	Puan; Zhouqin	Promoting blood flow and tendon relaxation	Medicinal liquor	SD-190	
*Veronicastrum caulopterum* (Hance) Yamazaki	Plantaginaceae	Ma xi lian	Si fang ma	Whole plant	Herb	Whole county	Heat-clearing and detoxifying	Decoction	SD-276	
*Viburnum cylindricum* Buch.-Ham. ex D. Don	Adoxaceae	Mei shi yang	Shui hong mu	Root; leaf; flower	Tree	Dujiang	Drainage of pus and dissolving carbuncle	Medicated bath	SD-354	
*Viburnum dilatatum* Thunb.	Adoxaceae	Yin lao	Jia mi	Root; leaf; fruit	Shrub	Whole county	Appetizing digestion; deficiency of Body Fluids	Grinding, decoction; orally soup	SD-493	
*Vicia sepium* L.	Fabaceae	Duo guo ni	Ye wan dou	Whole plant	Herb	Dahe; Sanhe	Inducing diuresis for removing edema	Decoction	SD-071	
*Vigna angularis* (Willd.) Ohwi et Ohashi	Fabaceae	Hong chi xiao dou	Chi dou	Seed	Herb	Whole county	Inducing diuresis for removing edema	Grinding, decoction; orally soup	SD-128	*
*Vincetoxicum atratum* (Bunge) Morren et Decne.	Apocynaceae	Mei bao fa	Bai wei	Root	Herb	Sanhe; Zhouqin	Moistening lung for arresting cough	Grinding, decoction; orally soup	SD-304	
*Vincetoxicum pycnostelma* Kitag.	Apocynaceae	Liao diao zhu	Xu chang qing	Whole plant	Herb	Dujiang; Zhouqin	Regulate the menstrual function to stop pain	Grinding, decoction; orally soup	SD-154	*
*Viola grypoceras* A. Gray	Violaceae	Ma kui lang	Zi hua jin cai	Whole plant	Herb	Whole county	Knife wound	Fresh herbs are placed on the affected area	SD-238	
Viola philippica* Cav*.	Violaceae	Ma gu	Zi hua di ding	Whole plant	Herb	Whole county	Decreasing swelling to relieving pain	Decoction	SD-208	
*Viola tricolor* L.	Violaceae	Xing fu na	San se jin	Whole plant	Herb	Whole county	Killing parasites to relieve itching	Grinding, decoction; orally soup	SD-457	
*Vitex negundo* L.	Lamiaceae	Mei dang jing	Huang jing	Fruit; root	Shrub	Dujiang; Sanhe	Dysentery	Grinding, decoction; orally soup	SD-313	
*Wahlenbergia marginata* (Thunb.) A. DC.	Campanulaceae	Ma mei xiang	Lan hua shen	Whole plant	Herb	Whole county	Infantile malnutrition	Boiled with meat and drunk the soup	SD-250	
*Wikstroemia indica* (L.) C. A. Mey	Thymelaeaceae	Yao dang ya	Liao ge wang	Stem; leaf; root	Shrub	Dujiang; Dahe	Blood circulation	Medicinal liquor	SD-466	
*Woodwardia japonica* (L. f.) Sm	Blechnaceae	Gou ji	Gou ji jue	Rhizome	Herb	Whole county	Killing parasites to relieve itching	Decoction	SD-112	*
*Xanthium strumarium* L.	Asteraceae	Ma lu bo	Cang er	Stem; leaf	Herb	Whole county	Expelling wind-damp	Decoction	SD-245	
*Xylosma congesta* (Loureiro) Merrill	Euphorbiaceae	Zao zi shu	Zuo mu	Leaf	Shrub	Sanhe	Heat-clearing and detoxifying; bruises	Grinding, decoction	SD-501	*
*Yulania liliiflora* (Desr.) D. L. Fu	Magnoliaceae	Mei ge de	Zi yu lan	Flower	Tree	Dujiang; Dahe	Dispelling wind and eliminating dampness	Grinding, decoction; orally soup	SD-334	
*Zanthoxylum bungeanum* Maxim	Rutaceae	Mei xiu	Hua jiao	Fruit	Tree	Whole county	Killing parasites to relieve itching	Grinding, decoction; orally soup	SD-359	
*Zanthoxylum dimorphophyllum* Hemsl	Rutaceae	Bai gei ga	Yi ye hua jiao	Leaf; root	Shrub	Dujiang; Jiuqian	Bruises; heat-clearing and detoxifying	Decoction	SD-014	
*Zanthoxylum nitidum* (Roxb.) DC.	Rutaceae	Mei xiu	Liang mian zhen	Rhizome; leaf	Shrub	Whole county	Dispelling wind and eliminating dampness	Boiled with meat and drunk the soup	SD-360	
*Zanthoxylum simulans* Hance	Rutaceae	Qing hua jiao	Ye hua jiao	Fruit; leaf; root	Shrub	Whole county	Harmonizing stomach; appetizing digestion	Boiled with meat and drunk the soup	SD-394	*
*Zehneria japonica* (Thunberg) H. Y. Liu	Cucurbitaceae	Yao di duo	Ma bo er	Root; leaf	Herb	Dahe; Shuilong	Detumescence	Boiled with meat and drunk the soup	SD-468	
*Zingiber mioga* (Thunb.) Rosc	Zingiberaceae	Ye jiang	Rang he	Rhizome; flower	Herb	Dujiang; Shuilong	Analgesia	Decoction	SD-490	*

*This medicinal plant was firstly recorded in Shui medicinal plants

**Fig. 4:**
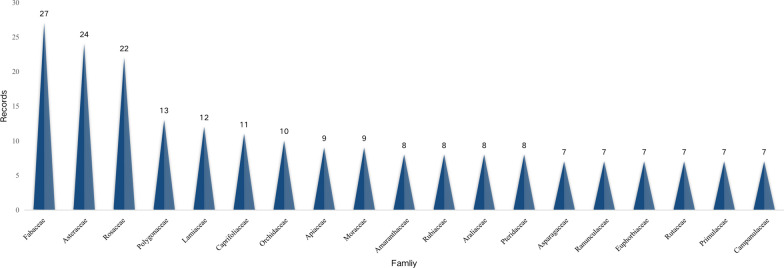
Nineteen prevalent botanical families with most species numbers used in Shui traditional medicine

The botanical families with the most medicinal plant species documented in this study are listed in Fig. 4. Fabaceae (27 species) and Asteraceae (24) families occupy the largest proportion of medicinal plants at this study site and are consistent with the wet monsoon climate. This is conducive to the survival of plants with more substantial regenerative and asexual reproduction [48]. Additional popular medicinal plant families include Rosaceae (22), Polygonaceae (13), Lamiaceae (12), Caprifoliaceae (11), Orchidaceae (10), Apiaceae (9), Moraceae (9), Amaranthaceae (8), Rubiaceae (8), Araliaceae (8), Pteridaceae (8), Asparagaceae (7), Ranunculaceae (7), Euphorbiaceae (7), Rutaceae (7), Primulaceae (7), and Campanulaceae (7). Similar results have been shown in Qiandongnan Miao and Dong Autonomous Prefecture, where many medicinal species belong to these families [40]. Although these 19 families accounted for 12.2% of the total number of families used by Shui healers, the number of species included accounted for 42.8% of the total number of medicinal species used, and of the remaining families, each contained only a few species.

Generally, the medicinal plant resources of the Shui ethnic group in Sandu County have three distinct characteristics:

1. Rich diversity: The findings highlight that the diverse natural environment of Sandu Shui Autonomous County provides local inhabitants with abundant natural resources. These medicinal plants not only help with disease prevention and treatment, but they provide a source of economic livelihood for locals. The rich biodiversity of this region can be credited to the forest protection and environmental traditions that the Shui people have developed and passed down through generations. While preserving forests and ecological environments, the Shui have fostered a symbiotic and mutually supportive relationship. For example, the Shui people frequently designate their revered mountains near their villages as “Dragon Mountains,” believing these mountains to be the foundation of their communities. The prosperity of the village is believed to be significantly impacted by the state of the Dragon Mountain, necessitating special protection measures, such as the prohibition of plant poaching and deforestation. Numerous forest protection and appreciation practices are also integrated into the Shui people's significant life-cycle ceremonies. When a baby boy is born in a Shui family, his father is obliged to plant a tree for the newborn and carefully tend to it, thereby symbolizing the child's growth and development. After a person passes away, *Liquidambar formosana* saplings are planted in their burial ground, and they are considered “divine trees.” Consequently, these *Liquidambar formosana* trees can thrive and form small “natural reserves” within the burial grounds. Even if these trees die or branches fall, their use for timber is strictly forbidden. The Shui people's proactive forest protection customs have effectively conserved local forest resources, paving the way for sustainable development in the region.
2. Widespread distribution and abundance: The villages where the Shui people reside are mostly located near mountains and water systems, such as Jiujian, Dahai, and Pu’an, helping to access more easily medicinal plants in the region. According to Shui healers, in the past, farmers from the Jiujian Township could obtain several thousand pounds of *Eucommia ulmoides* bark each time they collected in the mountains (unpublished results). After a thorough investigation, we observed the most commonly distributed medicinal plants included *Lonicera japonica*, *Ligustrum japonicum*, and *Houttuynia cordata*. Furthermore, each year during the Dragon Boat Festival, the Sandu county seat and its townships maintain a tradition of organizing a medicinal market, where vendors line both sides of the streets, creating a lively and bustling event. The Shui Dragon Boat Festival herbal market has played a significant role in the flourishing of the Chinese herbal medicine market in Sandu Shui Autonomous County. Besides the general public engaging in the buying and selling of herbal medicine, the county’s supply and marketing cooperatives, medical departments, and local produce departments also set up stalls at major intersections to purchase medicinal plants, such as *Platycodon grandiflorus*, *Asparagus cochinchinensis*, *Ophiopogon japonicu*s, and *Uncaria rhynchophylla*. Consequently, a substantial volume of herbal medicine is sold in Sandu annually, establishing it as one of the primary export products of the Sandu Shui Autonomous County. The herbal medicine industry in Sandu shows great potential for further growth and development.
3. Extensive cultivation: In order to make them more easily accessible for personal use, common medicinal plants, such as *Celosia cristata, Isodon amethystoides*, and *Asarum insigne*, have been cultivated around houses and on farmland for immediate use. In addition, some Shui people also grow edible wild plants with medicinal properties, like *Pteridium aquilinum*, *Houttuynia cordata*, and *Capsella bursa-pastori*s. The cultivation of these plants does not require time-consuming management or pesticide application, allowing them to retain their authentic flavor. This practice can be viewed as a transition from humans wildcrafting plants in their natural habitat to engaging in cultivation. Additionally, there is also commercial production through cultivation. At present, as farmers and the local government recognize the medicinal and economic value of Shui medicinal plants, Sandu County has initiated large-scale cultivation of medicinal plants such as *Eucommia ulmoides*, *Gastrodia elata*, and *Mahonia fortunei*. This approach has transformed medicinal plant resources into an economic powerhouse for the region, ensuring a steady supply of medicinal materials for businesses and providing an excellent income-generating opportunity for the local community.

### Traditional uses and preparation of medicinal plants

**Fig. 5:**
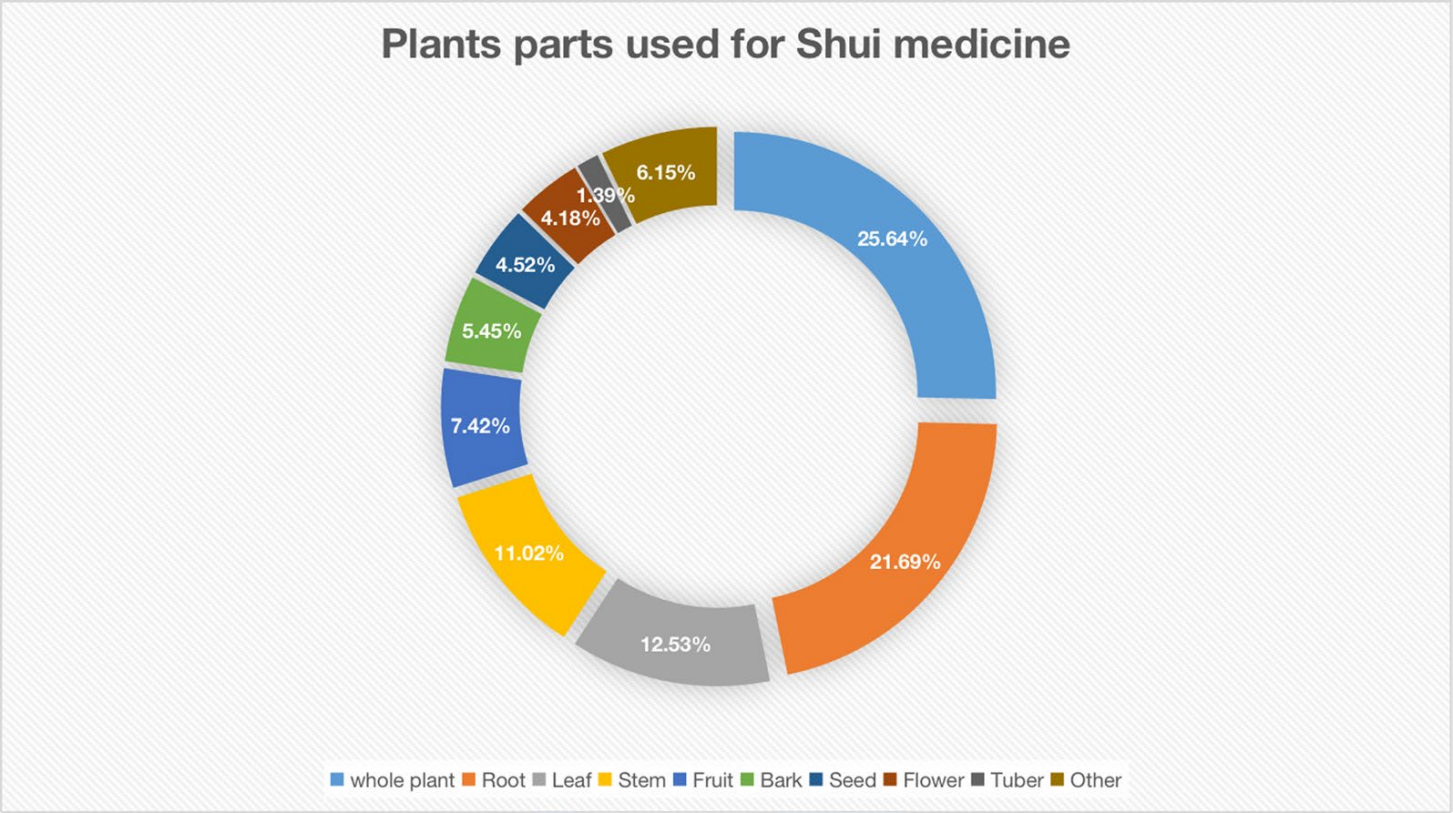
Plant parts used in Shui medicine

The efficacy of medicinal plants is closely related to the plant part used since different parts of the same plant may have different uses and effectiveness [49]. The Shui informants at the study sites used multiple parts of medicinal plants, including the root, whole plant, leaf, stem, bark, fruit, seed, flower, and tuber. Among them, the whole plants were the most popularly used group in terms of species number (221 species), accounting for 25.64% of the total species, followed by roots (21.69%), leaves (12.53%), stems (11.02%), fruits (7.42%), barks (5.45%), seeds (4.52%), flowers (4.18%), tubers (1.39%), and others, including bulbs, vine, and rhizome (6.15%) (Fig. 5).

Using the whole plants as medicine is common practice in many ethnic medicines, and traditional doctors believe that this method can enhance the efficacy of the medicine [50, 51]. Although this collection method can cause damage to the local medicinal plant resources and harm biodiversity conservation, it is worth noting that some villagers have taken to cultivating commonly used medicinal plants in their home gardens as an alternative. Furthermore, the county government has taken steps in recent years to initiate reforestation efforts and has implemented administrative measures to safeguard medicinal plant resources [52]. As a result, the issue of destructive collection and excavation has begun to be somewhat alleviated.

In all, 374 traditional medicine prescriptions were collected through interviews with local healers and included nine types of treatments: decoction (278); external application (34); medicinal liquor (36); oral soup (128), and exterior washing (28). The Shui people use fresh medicinal plants frequently, while dry plants are seldom used. This is because the Shui believe the active ingredients of fresh plants are still intact so that this method can optimize effectiveness. However, our investigations found that most plants traded at the market were dried. Local herbal medicine vendors explained that these dried medicinal materials were more convenient for storage. In addition, dried plant materials also were considered to have improved taste and odor, so they are more palatable.

Medicinal liquor, for the prevention and treatment of diseases, is one of the oldest traditional dosage forms in the history of Chinese medicine. These liquors have been widely used in both folk and medicinal industries from ancient times to the present day [53–55]. Shui people prepare alcoholic beverages, known in Chinese as *jiuqian-jiu*, which are made from rice and special starter made of wild plants known as *jiuqu* [56, 57]. This dosage form has antiseptic and antitoxic properties, which can delay hydrolysis and enhance the stability of many medicines [58]. There are 36 medicinal plant species used for both medicine and *Jiuqian* liquor starters (Table 6). For example, *Lygodium japonicum* is used to treat urinary tract infections, hepatitis, nephritis edema, and diarrhea, while the Miao people stew it with meat to strengthen their constitution [40]. *Melastoma dodecandrum* is used traditionally for expelling wind-damp. A few species are used for both food and medicine, such as *Imperata cylindrica* and *Rosa roxburghii.*

**Table 6:** Plants used for both medicine and Jiuqian liquor starters

Scientific name	Chinese name (pin yin)	Shui name	Part used as starter	Part used as medicine	Medicinal use and value
*Agrimonia pilosa* Ledeb.	Long ya cao	Ma ban bie	Whole plant	Whole plant; root	Dysentery; bleeding
*Ainsliaea fragrans* Champ.	Xing xiang tu er feng	Pa zheng	Whole plant	Whole plant	Bruises; heat-clearing and detoxifying
*Ardisia japonica* (Thunb.) Blume	Zi jin niu	Za du	Aerial part	Stem; root	Hemostasis; bruises
*Asplenium trichomanes* L.	Tie jiao jue	Gang du gun	Whole plant	Whole plant	Drainage of pus and dissolving carbuncle
*Bletilla striata* (Thunb. ex Murray) Rchb. F.	Bai ji	Gang jie ba	Root	Tuber	Strengthen the spleen
*Brandisia hancei* Hook. F.	Lai jiang teng	Ma miao	Whole plant	Whole plant	Dysentery; dispelling wind and eliminating dampness
*Cunninghamia lanceolata* (Lamb.) Hook.	Shan	Mei ao	Leaf	Bark; root; leaf	Dispelling wind and eliminating dampness
*Duhaldea cappa* (Buchanan-Hamilton ex D. Don) Pruski & Anderberg	Yang er ju	Ma pang da	Aboveground part	Whole plant; root	Regulating the menstrual function to stop pain; eliminating cold stop pain
*Elaeagnus pungens* Thunb.	Hu tuo zi	Mei du	Fruit, leaf	Fruit; root; leaf	Antitussive; eliminating phlegm and stopping cough
*Gardenia jasminoides* Ellis	Zhi zi	Mei le	Leaf, fruit	Fruit	Blood cooling and arresting; heat-clearing and detoxifying
Gerbera piloselloides (L.) Cass.	Tu er yi zhi jian	Ba hao	Whole plant	Whole plant	Eliminating phlegm and stopping cough; moistening lung for arresting cough
*Glochidion puberum* (L.) Hutch.	Suan pan zi	Mei nv ban	Stem, leaf, fruit	Fruit	Antidiarrheic
*Gonostegia hirta* (Bl.) Miq.	Nuo mi tuan	Ma ao xing	Whole plant	Whole plant	Heat-clearing and detoxifying; clearing heat; dehumidification
*Hedera nepalensis* K. Koch	Chang chun teng	Ma lian man	Whole plant	Stem; leaf	Removing pathogenic heat from the blood and toxic material from the body; dispelling wind and eliminating dampness
*Hypericum japonicum* Thunb. ex Murray	Di er cao	Ma ka di	Aboveground part	Whole plant	Hepatitis
*Hypericum sampsonii* Hance	Yuan bao cao	Ma suan long	Whole plant	Whole plant	Bruises
*Imperata cylindrical* (L.) Beauv	Bai mao	Gang yao man	Rhizome	Root; leaf; flower	Removing pathogenic heat from the blood and toxic material from the body
*Leonurus japonicus* Houttuyn	Yi mu cao	Ma ka bo	Stem, leaf	Whole plant; seed	Regulating the menstrual function to stop pain
*Lilium brownii* F. E. Brown ex Miellez	Ye bai he	Qiu ba	Bulb	Tuber	Moistening lung for arresting cough
*Lophatherum gracile* Brongn.	Dan zhu ye	Tu wa fen	Whole plant	Whole plant	Removing pathogenic heat from the blood and toxic material from the body
*Lygodium japonicum* (Thunb.) Sw.	Hai jin sha	Mao nu ga	Whole plant	Root; stem	Inducing diuresis for removing edema
*Melastoma dodecandrum* Lour.	Di ren	Ma geng	Whole plant	Whole plant	Expelling wind-damp
*Morella rubra* Lour.	Yang mei	Ma kang	Leaf, fruit	Root	Hemostasis
*Nephrolepis cordifolia* (L.) C. Presl	Shen jue	ni ge ding	Whole plant	Tuber; leaf	Eliminating phlegm and stopping cough
*Paederia foetida* L.	Ji shi teng	Yao de ma	Whole plant	Whole plant	Invigorates the spleen and promotes digestion
*Platycodon grandiflorus* (Jacq.) A. DC.	Jie geng	Xiang dian	Root	Root	Eliminating phlegm and stopping cough
*Polygala japonica* Houtt	Gua zi jin	Dong yao dong	Aboveground part	Whole plant; root	Eliminating phlegm and stopping cough
*Portulaca oleracea* L.	Ma chi xian	Ma wa fa	Aboveground part	Whole plant	Dysentery
*Pueraria montana* var. *lobata* (Willdenow) Maesen & S. M. Almeida ex Sanjappa & Predeep	Ge	Yao hai	Leaf	Tuber	Eczema; dispelling wind and eliminating dampness
*Rohdea japonica* (Thunb.) Roth	Wan nian qing	Gang ao mie	Leaf	Whole plant; root	Harmonizing stomach; appetizer digestion
*Rosa laevigata* Michx	Jin ying zi	Dou pang ya	Leaf, fruit	Fruit; root	Tonifying kidney
*Rosa roxburghii* Tratt.	Sao si hua	Pang ka	Leaf	Fruit; root	Harmonizing stomach
*Sargentodoxa cuneata* (Oliv.) Rehd. et Wils.	Da xue teng	Yao e nong	Rhizome	Root; stem	Strong bones and muscles
*Toona sinensis* (A. Juss.) Roem	Xiang chun	Mei han ga	Leaf	Bark	Dispelling wind and eliminating dampness
*Verbena officinalis* L.	Ma bian cao	Ma ou	Aboveground part	Stem	Active blood and disperse stagnation
*Zanthoxylum bungeanum* Maxim.	Hua jiao	Mei xiu	Leaf	Fruit	Killing parasites to relieve itching

Although Shui medicine is based on the principles of male progenitor lineage, women are primarily responsible for brewing *Jiuqian-jiu*. The Shui believe that medicinal plants should be harvested around the Dragon Boat Festival to achieve the best curative effect. Therefore, on the morning of the Dragon Boat Festival, the experienced elder female team leader from a village leads other women up the mountains to harvest and clean the medicinal herbs. The team leader then turns the collected herbs into medicinal liquor, which is not easy to prepare, usually taking at least 3 months for fermentation. After the medicinal liquor has been cured, the team leader distributes it to each household, and the women mix it with steamed glutinous rice. After further fermentation, the sweet and nutritious *Jiuqian*-*jiu* is ready to drink. All villages in the Shui region participate in alcohol brewing. Sweet rice wine cooked with eggs is a vital source of nourishment for women during their postpartum period. In some villages, there is a tradition of sealing a bottle of freshly brewed alcohol upon a baby's birth, only to be unsealed when the child gets married or passes away, thereby serving as a way to honor ancestors and entertain guests. The most renowned liquor is *Jiuqian* wine, which has earned a reputation as a widely celebrated specialty.

In addition to medicinal liquor, the Shui often use their secret recipes for healing. For example, when a child’s bone is fractured, it can be fixed with a small splint made of *Gonocarpus micranthus* for 2 days and then wrapped in a poultice prepared with *Sargentodoxa cuneata, Schisandra chinensis*, and *Eucommia ulmoides* for 5 days. Finally, the broken limb is washed with a decoction of *Sambucus williamsii*, *Dichondra micrantha*, *Rhus chinensis*, *Ficus tikoua*, *Sargentodoxa cuneata*, and *Heptapleurum heptaphyllum*. This traditional medical practice involves the use of a number of species instead of a single herb, a common practice of Shui healers.

Although there are various forms of traditional medicine used by the Shui people, including soaking the plants in water to treat ailments like colds, coughs, diarrhea, and hemoptysis, or soaking them in alcohol to treat injuries and rheumatism, as well as using water or alcohol for external application to treat snake bites, insect bites, fractures, and cuts, there are not many other formulations, like ointments, pills, or powders. During treatment, patients are typically asked about their symptoms, but there are no standardized measurements or preparation methods. Thus, Shui traditional medicine is still at the early stage of experience-based treatment, awaiting a transition from empirical to theoretical knowledge, and the elevation of experience to theory.

### Diseases treated and characteristics of Shui medicine

**Fig. 6:**
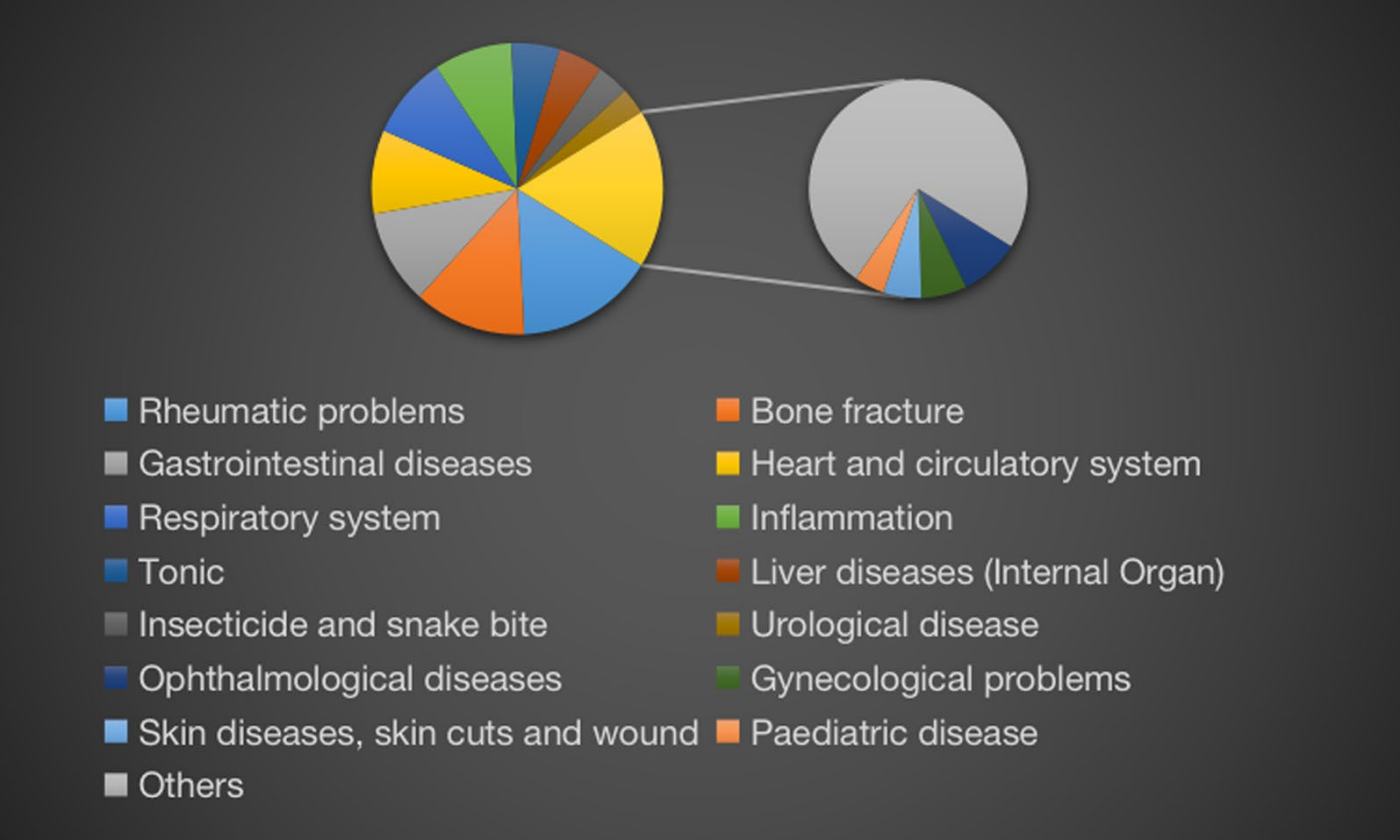
Major functions of Shui medicinal plants

Shui medicinal plants are used to treat 85 human ailments [59]. The most prevalent ailments treated with documented medicinal plants are rheumatic diseases (78, 15.45%), bone fractures (63, 12.48%), gastrointestinal system diseases (53, 10.50%), heart and circulatory system (47, 9.31%), respiratory diseases (46, 9.11%), inflammation (44, 8.71%), tonic (27, 5.35%), liver diseases (25, 4.95%), insecticide and snake bite (18, 3.56%), urological diseases (15, 2.97%), ophthalmological diseases (8, 1.58%), gynecological problems (6, 1.19%), skin diseases (5, 0.99%), pediatric disease (4, 0.79%), and others (66, 13.07%) (Fig. 6).

Disease incidence is often closely related to the local environment and climate, as well as ethnic activities and lifestyles [41]. According to the survey, Sandu had the largest number of healers who could effectively treat rheumatic disease, and this may be because Sandu is located in the Moon and the Leigong Mountains. This area is mountainous, with dense forests, high temperatures, rainy weather, wind, cold, and damp heat, and these climatic conditions are conducive to developing rheumatism. From the theory of traditional Chinese medicine, those who live in damp areas should expel wind from their body regularly to relieve constipation and improve their sleep quality, thereby improving their health [60]. Thus, due to their unique environment, Shui people have identified many herbs to treat rheumatism.

Moreover, herbs to treat fractures comprise a large proportion of Shui traditional medicines (Fig. 6). Bone fractures are an occupational hazard for local people engaged in agriculture and forestry. For example, a Shui healer may treat bone fractures with poultices prepared from fresh flowers and bark of *Albizia julibrissin*, *Prunella vulgaris*, and *Gonostegia hirta*. Also, a chicken's internal organs are removed and the chicken is filled with freshly macerated *Reineckea carnea* for external application to a fractured bone. During interviews with a Shui healer in Dahe Township, we learned that this method was used to successfully treat over 20 patients with bone fractures, with highly effective outcomes.

### Popularity of medicinal plants and other health-promoting customs

The RFC (relative frequency of citation) was adopted to evaluate important plant species used by local healers to treat various diseases. From the 374 prescriptions investigated, the number of prescriptions mentioning plant species (FC) used ranged from 1 to 15. Calculations showed that 12 medicinal plant species had FC > 10 (Table 7). The RFC values calculated for these 12 medicinal plant species ranged from 0.027 to 0.041. The medicinal plants with higher RFC values included *Isodon amethystoides*, *Asarum insigne*, and *Acorus tatarinowii*.

**Table 7:** Relative frequency of citation (RFC) of plant species mentioned in prescriptions, from high to low RFC

Scientific name	FC	RFC	Scientific name	FC	RFC
*Acorus tatarinowii*	15	0.041	*Asparagus cochinchinensis*	12	0.031
*Asarum insigne*	15	0.041	*Rosa laevigata*	12	0.031
*Isodon amethystoides*	15	0.041	*Allium macrostemon*	11	0.029
*Aster indicus*	14	0.037	*Valeriana jatamansi*	11	0.029
*Chrysanthemum indicum*	14	0.037	*Bletilla striata*	10	0.027
*Gleditsia sinensis*	13	0.034	*Pueraria montana* var. *lobata*	10	0.027

The high RFC values in this study highlight the local healers and residents have a strong dependence on these 12 species of medicinal plants (Table 7). The higher the RFC value, the more familiar the local healers are with the species. Furthermore, and of great importance, these species were either highly effective or abundant and easy to obtain locally. Because of their popularity in Shui medicine, all of these plants should be further studied, focusing on their chemistry, pharmacology, and toxicity, as well as evaluation of the efficacy and safety of local medicinal plants.

For example, *Isodon amethystoides* is widespread throughout Sandu County and is well known among the Shui people for its medicinal properties. Local traditional Shui healers use the whole plant or root to treat the cancer, autoimmune diseases, and other difficult-to-cure diseases. Compared with some Western medicines, *Isodon amethystoides* has significantly fewer side effects and can improve the body’s immunological function [61]. With the trend of using naturally occurring substances, drugs from plants have become increasingly important alternative medicines worldwide [62, 63]. *Isodon amethystoides* deserves further study for drug development.

*Asarum insigne*, a common substitute for *Asarum heterotropoides,* has significant pharmacological action, strong therapeutic effects, and easily sourced. In Sandu, it is widely used for the treatment of windchill pain, toothache, broken bones, snake bites, acute gastroenteritis, bacillary dysentery, windchill cough, windchill cold, chronic bronchitis, asthma, and chronic gastritis. Recent experiments have shown that the whole plant of *Asarum insigne* contains various amino acids and inorganic elements, and has anti-aging, blood pressure- and lipid-lowering effects [64]. Some researchers have processed it to make it more palatable, with a slightly floral aroma [65]. It is believed that through the application of state-of-the-art instruments such as high performance liquid chromatography-mass spectrometry, the effective pharmacological active components of *Asarum insigne* can be elucidated and it has the potential to be developed into an externally applied medicine.

*Acorus tatarinowii* is also an important Chinese medicinal material, which is used in the clinical treatment of forgetfulness, tinnitus, deafness, rheumatism, and pain [66]. The growth cycle of *Acorus tatarinowii* is typically 3–4 years, and its regeneration rate is slow after excessive harvesting. Thus, *Acorus tatarinowii* resources are in short supply because of the destruction of its natural environment, and thus the price of wildcrafted *Acorus* plants has been increasing in recent years. Wild *Acorus tatarinowii* resources are mainly found in remote mountains, and harvesting has become more difficult since young Shui men have been abandoning rural villages for better jobs in urban areas (Table 4). Harvesting wild *Acorus* is mainly left to some older farmers, and this increases the labor cost, resulting in more expensive *Acorus tatarinowii,* especially compared with other medicinal herbs.

The Shui people have various customs in their daily life that are closely related to their health. For instance, during festivals, they use *Paederia foetida* to make rice cakes, and they create “Hui Zong Ba” by mixing glutinous rice with burned rice straw ash, which is rich in calcium, potassium, and other essential elements that supplement their health [65]. As a substitute for tea, the Shui people often drink *Ligustrum japonicum*, which has the beneficial effects of clearing heat and detoxifying the body. Additionally, *Pseudognaphalium affine*, a popular wild vegetable for Shui people, has the property of relieving coughs and reducing phlegm (Table 5). The Shui people use *Strobilanthes cusia* to dye their traditional clothing, which has a therapeutic effect that translates from Chinese to English as “clothing therapy” [66].

*Paederia foetida*, commonly known as “Jishiteng” in Chinese, has leaves that emit a distinct odor resembling chicken feces when crushed. However, this plant is believed to have medicinal properties that nourish *yin* and strengthen *yang*, invigorate *qi* and blood in the Shui community (Table 5). Glutinous rice cultivation has a long history among Shui communities, who have developed many methods of processing it. One of their favorite delicacies is a steamed cake made by mixing *Paederia foetida* with glutinous rice. To prepare this dish, glutinous rice is soaked in water for 3–4 h, while the freshly picked *Paederia foetida* leaves are cleaned, chopped and the juice is extracted through a cheesecloth. The extracted juice is then mixed with glutinous rice powder. A pot of water is brought to boil and sugar is added until it dissolves. The water with sugar is then poured into the glutinous rice and *Paederia foetida* juice mixture, stirring until evenly distributed. A steaming dish is greased and the mixture is poured in, then steamed in layers until fully cooked. The result is a tasty and fragrant *Paederia foetida* glutinous rice cake that is considered beneficial to health. Adding glutinous rice and sugar not only eliminates the odor of *Paederia foetida*, but also imparts a pleasant fragrance to the dish.

The Shui people weave their own clothing and traditionally dye it blue using indigo, *Strobilanthes cusia*. Even today, those living in rural areas continue to favor indigo-dyed clothes. The Shui often engage in outdoor labor, frequently scratching their hands and feet, so wearing indigo clothes may help to prevent wound infections and alleviate skin itching (Table 5).

## Conclusion

The Sandu region boasts abundant medicinal plant resources, and the Shui people have a long-standing tradition of utilizing these plants to treat various ailments in their daily lives. In this study, we analyzed the data collected from 15 healers and another 132 informants who used fresh or dried herbal medicinal material of 505 plant species to treat a wide spectrum of illnesses and diseases, which belong to 405 genera from 156 families, with Fabaceae being the highest represented plant family. Most of the Shui medicinal plants are herbaceous, and healers most commonly use the whole plants in their treatments. Of the 85 different diseases treated by these medicinal plants, a significant number were to treat rheumatism and bone fractures, which may correlate with the local living and environmental conditions. The local people commonly used three medicinal plant species: *Isodon amethystoides*, *Asarum insigne*, and *Acorus tatarinowii*. Further studies on their chemistry, biological activity, and toxicity are needed for potentially developing new pharmaceutical products.

Based on field investigations, this study has comprehensively collected, organized, analyzed, evaluated, and summarized the medicinal plant resources and associated traditional knowledge developed and utilized by the Shui people. The results provide strong scientific evidence for the future development, utilization, and protection of Shui medicine. However, it is important to acknowledge that traditional medicinal knowledge and medicinal plants face great threats from rapid urbanization.

For instance, Jiuqian Town had the highest per capita distribution of healers (Pch), only one local healer was in Zhonghe Township. Of the 15 local healers surveyed in this study, only two were younger than 40 years old. Men and older healers with less education possess most of the knowledge regarding herbal remedies. Meanwhile, most younger people prefer to look for jobs in urban areas instead of studying traditional medicinal knowledge in the countryside. Thus, there is an urgent need to implement policies and practices for the conservation of medicinal plants and their associated traditional knowledge. This will ensure that this valuable knowledge is not lost to future generations.

## Data Availability

All data generated or analyzed during this study are included in this published article.
